# Multitopic 3,2′:6′,3′′-terpyridine ligands as 4-connecting nodes in two-dimensional 4,4-networks†

**DOI:** 10.1039/d2ce01130a

**Published:** 2022-09-23

**Authors:** Giacomo Manfroni, Bernhard Spingler, Alessandro Prescimone, Edwin C. Constable, Catherine E. Housecroft

**Affiliations:** Department of Chemistry, University of Basel Mattenstrasse 24a, BPR 1096 4058-Basel Switzerland catherine.housecroft@unibas.ch; Department of Chemistry, University of Zurich Winterthurerstr. 190 8057-Zurich Switzerland

## Abstract

The tetratopic 1,4-bis(2-phenylethoxy)-2,5-bis(3,2′:6′,3′′-terpyridin-4′-yl)benzene (1) and 1,4-bis(3-phenylpropoxy)-2,5-bis(3,2′:6′,3′′-terpyridin-4′-yl)benzene (2) ligands have been prepared and fully characterised. Combination of ligand 1 or 2 and [M(hfacac)_2_]·*x*H_2_O (M = Cu, *x* = 1; M = Zn, *x* = 2) under conditions of crystal growth by layering led to the formation of [Cu_2_(hfacac)_4_(1)]_*n*_·3.6*n*(1,2-Cl_2_C_6_H_4_)·2*n*CHCl_3_, [Zn_2_(hfacac)_4_(1)]_*n*_·*n*MeC_6_H_5_·1.8*n*CHCl_3_, [Cu_2_(hfacac)_4_(2)]_*n*_·*n*MeC_6_H_5_·2*n*H_2_O, [Cu_2_(hfacac)_4_(2)]_*n*_·2.8*n*C_6_H_5_Cl and [Cu_2_(hfacac)_4_(2)]_*n*_·2*n*(1,2-Cl_2_C_6_H_4_)·0.4*n*CHCl_3_·0.5*n*H_2_O. For each compound, single-crystal X-ray analysis revealed the assembly of a planar (4,4)-net in which the tetratopic ligands 1 or 2 define the nodes. The metal centres link two different bis(3,2′:6′,3′′-tpy) ligands *via* the outer pyridine rings; whereas copper(ii) has N-donors in a *trans*-arrangement, zinc(ii) has them in *cis*. This difference between the copper(ii) and zinc(ii) coordination polymers modifies the architecture of the assembly without changing the underlying (4,4)-network.

## Abstract 2

Ligands containing two 3,2′:6′,3′′-terpyridine metal-binding domains act as 4-connecting nodes in 2D (4,4)-networks; subtle structural changes occur on coordination to Cu(ii) or Zn(ii).

## Graphical abstract

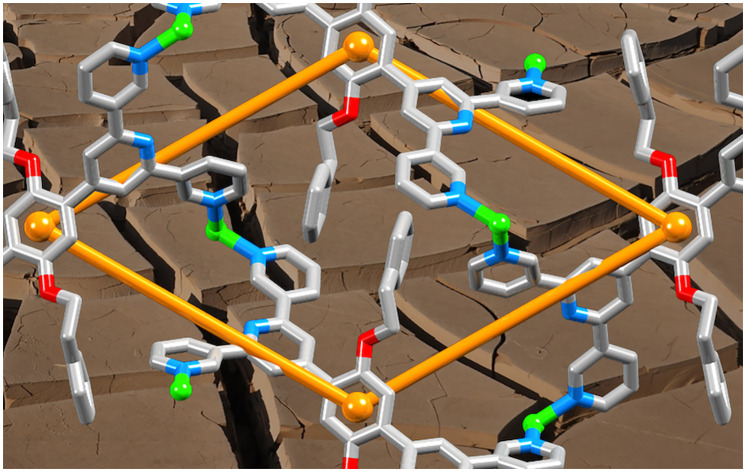

## Introduction

**Scheme 1:**
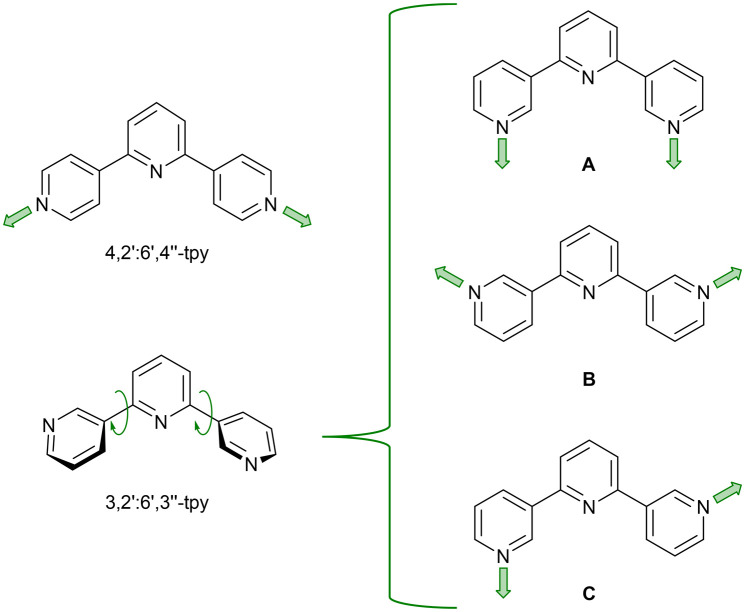
Divergent binding mode of 4,2′:6′,4′′-tpy, and some of the possible binding modes (labelled A–C) that 3,2′:6′,3′′-tpy can adopt *via* inter-ring C–C bond rotation. The two tpy isomers coordinate only through the outer N atoms.

The symmetric, divergent and isomeric ligands, 4,2′:6′,4′′-terpyridine (4,2′:6′,4′′-tpy) and 3,2′:6′,3′′-terpyridine (3,2′:6′,3′′-tpy) are well-suited for use as building blocks in supramolecular chemistry,^1^ with their ability to participate in π-stacking and directional hydrogen-bonding interactions in addition to binding metal centres. Their 4′-aryl functionalized derivatives are readily prepared using the one-pot Hanan^2^ procedure, which is based on the general method of Kröhnke,^3^ although there are some serendipitous instances in which cyclic products are favoured.^4^ Unlike 2,2′:6′,2′′-tpy, which typically coordinates as a tridentate κ^3^ chelating unit, the central pyridine ring is non-coordinating in 3,2′:6′,3′′-tpy and 4,2′:6′,4′′-tpy, and typically, they behave as ditopic *N*,*N*′-donor ligands. These two tpy isomers possess different degrees of coordination flexibility (Scheme 1). In 4,2′:6′,4′′-tpy, inter-ring C–C bond rotation does not affect the orientation of the *N*,*N′*-donor set, leading to a V-shaped building block. In contrast, conformational changes (Scheme 1) determine the directionality of the *N*,*N′*-donor set in 3,2′:6′,3′′-tpy leading to a more versatile and less predictable network assembly.^5^

**Scheme 2:**
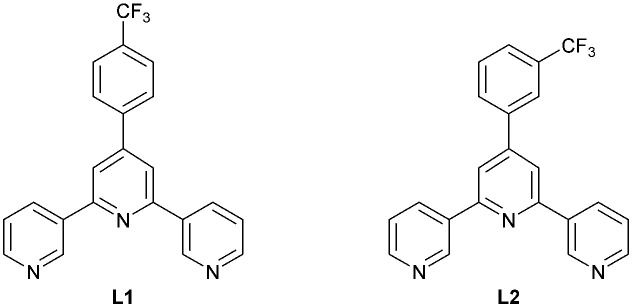
The structures of ligands L1 and L2.^26^

Although many examples of 1D-, 2D- and 3D-assemblies have been prepared from ligands containing one or more 4,2′:6′,4′′-tpy units, the coordination behaviour of 3,2′:6′,3′′-tpy ligands remains less exploited.^1,5–25^ We have reported three copper(ii) 1D-coordination polymers [Cu_2_(hfacac)_4_(L1)_2_]_*n*_·*n*(1,2-Cl_2_C_6_H_4_) (Hhfacac = 1,1,1,5,5,5-hexafluoropentane-2,4-dione), [Cu_2_(hfacac)_4_(L1)_2_]_*n*_·*n*C_6_H_5_Cl, and [Cu(hfacac)_2_(L2)]_*n*_·*n*C_6_H_5_Cl, containing ditopic 3,2′:6′,3′′-tpy ligands with coordinatively innocent 4′-substituents. In [Cu_2_(hfacac)_4_(L1)_2_]_*n*_·*n*(1,2-Cl_2_C_6_H_4_) and [Cu_2_(hfacac)_4_(L2)_2_]_*n*_·*n*C_6_H_5_Cl the 3,2′:6′,3′′-tpy domains exhibit conformation **C**, while with [Cu(hfacac)_2_(L2)]_*n*_·*n*C_6_H_5_Cl conformation **B** is adopted (Scheme 1).^26^ Structures of ligands L1 and L2 are shown in Scheme 2.

**Scheme 3:**
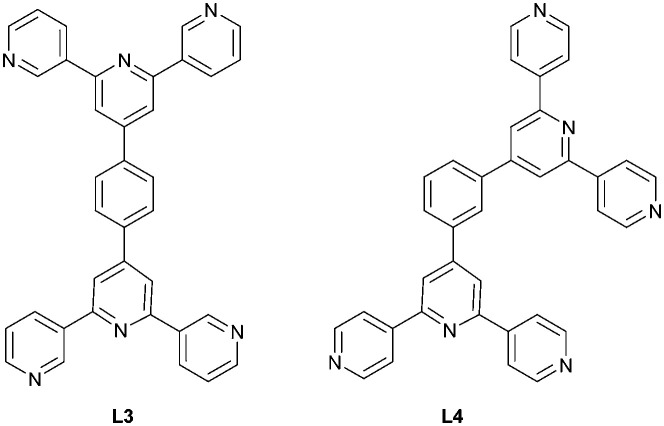
Structures of the tetratopic bis(tpy) ligands L3 and L4.

One strategy for increasing the dimensionality of an assembly is to select metal centres which favour higher coordination numbers combined with ditopic 3,2′:6′,3′′-tpy or 4,2′:6′,4′′-tpy linkers. The resulting coordination network is thereby directed by the metal node.^5,27^ An alternative methodology to encourage the formation of 2D- and 3D-dimensional assemblies is by connecting multiple tpy domains to appropriate scaffolds. Two 3,2′:6′,3′′-tpy or 4,2′:6′,4′′-tpy units can be linked in a “back-to-back” fashion by any organic spacer, generating a tetratopic ligand.^8,28^ We decided to extend our investigations of [M(hfacac)_2_] coordination chemistry to tetratopic bis(tpy) ligands, and we recently demonstrated the assembly of a series of (4,4) nets based on 1,4-bis(*n*-alkyloxy)-2,5-bis(3,2′:6′,3′′-terpyridin-4′-yl)benzene ligands.^29^ A search of the Cambridge Structural Database (CSD v. 2021.3.0, April 2022) revealed only three other structures involving a bis(4,2′:6′,4′′-tpy) or bis(3,2′:6′,3′′-tpy) with a metal 1,3-diketonate. Yoshida *et al.* showed that ligand L3 (Scheme 3) combined with [Co(acacCN)_2_] (HacacCN = 2-acetyl-3-oxobutanenitrile) gives the 2D-dimensional (4,4) net [Co_2_(acacCN)_4_(L3)]_*n*_. The replacement of [Co(acacCN)_2_] by [Co(dbm)_2_] (Hdbm = 1,3-diphenylpropane-1,3-dione) leads to a 1D-chain [Co(dbm)_2_(L3)]_*n*_, in which L3 is bidentate through one pyridine N-donor from each tpy domain. The low connectivity is perhaps sterically induced by the presence of larger phenyl rings in [Co(dbm)_2_]. In contrast, in [Co_2_(acacCN)_4_(L4)]_*n*_, a change in the ligand from L3 to L4 (Scheme 3) with [Co(dbm)_2_] does not change the topology of the coordination network, but the networks exhibit 3-fold interpenetration in the solid state structure.^25^

**Scheme 4:**
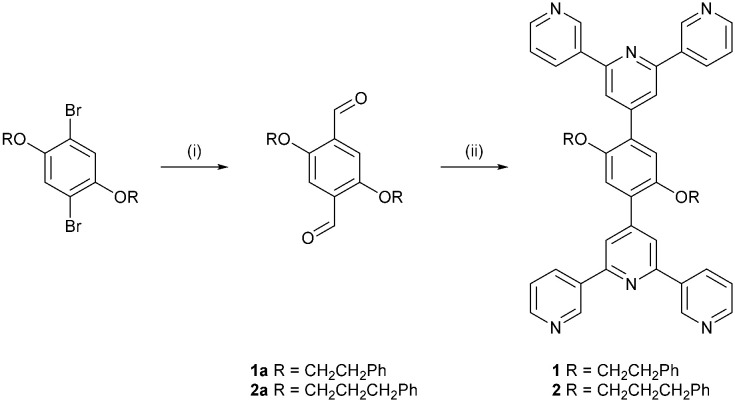
Synthetic route to 1 and 2. Reagents conditions (see ESI† for full details): (i) *n*-BuLi, Et_2_O, 0 °C; dry DMF, warmed to room temperature, 22 h; (ii) 3-acetylpyridine, KOH, NH_3_, EtOH, RT, 6 days for 1 and 5 days for 2.

In this work, we report the synthesis of two bis(3,2′:6′,3′′-tpy) ligands 1 and 2 (Scheme 4) with 1,4-phenylene spacers containing 2-phenylethoxy and 3-phenylpropoxy substituents attached to the phenylene moiety. We have already demonstrated that introducing alkyloxy groups enhances the solubility of the ligand in organic solvents which is beneficial for crystal growth. In addition, the nature of the alkyloxy group can influence the assembly and if terminal phenyl groups are present, they may participate in π–π stacking interactions within the solid state.^5,8,28^ Herein, we describe the reaction of 1 and 2 (potentially tetratopic ligands) with [M(hfacac)_2_] (M = Cu, Zn), a two connecting building block, to generate a series of 2-dimensional (4,4)-networks. The electron-withdrawing effect of the CF_3_ substituents improves the affinity of the complex towards coordination with the pyridine donors of the tetratopic ligands as well as improving its solubility in organic solvents.

## Experimental

Materials and full synthetic procedures are given in the ESI,† including characterization. Crystallographic data are given in Tables 1 and 2.

**Table 1:** Crystallographic data of the dialdehydes 1a and 2a

Compound	1a	2a
Empirical formula	C_24_H_22_O_4_	C_26_H_26_O_4_
Formula weight	374.41	402.47
Crystal system	Monoclinic	Orthorhombic
Space group	*P*2_1_/*n*	*Pbca*
*a* [Å]	12.4540(7)	9.0988(6)
*b* [Å]	5.4805(2)	8.7410(6)
*c* [Å]	14.1111(7)	27.1654(17)
*α* [°]	90	90
*β* [°]	104.263(4)	90
*γ* [°]	90	90
*V* [Å^3^]	933.45(8)	2160.5(2)
*Z*	2	4
*D*_c_ [g cm^−3^]	1.332	1.237
*T* [K]	150	150
Wavelength [Å]	1.34143	1.54178
*μ* [mm^−1^]	0.462	0.661
*F*(000)	396	856
Crystal size [mm^3^]	0.3 × 0.2 × 0.1	0.25 × 0.20 × 0.08
Crystal description	Yellow prism	Yellow plate
*θ* range (data collect.) [°]	3.694 to 56.730	5.853 to 69.871
Index ranges	−15 ≤ *h* ≤ 14, −4 ≤ *k* ≤ 6, −17 ≤ *l* ≤ 16	−10 ≤ *h* ≤ 10, −10 ≤ *k* ≤ 9, −32 ≤ *l* ≤ 32
Measured Refl's.	5213	14 033
Indep't Refl's	1854	1995
*R*_int_	0.0802	0.0334
Refl's *I* > 2*σ* (*I*)	1614	1817
Completeness to *θ*	98.7% to 53.597°	99.6% to 67.679°
Redundancy	2.81	7.03
Absorption correction	Multi-scan	Multi-scan
Max. and min. transmission	0.218 and 0.000	0.753 and 0.695
Data/restraints/parameters	1854/0/127	1995/0/136
Gof on *F*^2^	1.258	1.015
*R*_1_ [*I* > 2*σ* (*I*)]	0.1118	0.0354
w*R*_2_ [*I* > 2*σ* (*I*)]	0.2784	0.0950
*R*_1_ all data	0.1152	0.0386
w*R*_2_ all data	0.2849	0.0984
Largest diff. peak and hole [e Å^−3^]	0.720 and −0.565	0.198 and −0.160
CCDC	2162893	2162895

**Table 2:** Crystallographic data of the coordination polymers [Cu_2_(hfacac)_4_(1)]_*n*_·3.6*n*(1,2-Cl_2_C_6_H_4_)·2*n*CHCl_3_, [Zn_2_(hfacac)_4_(1)]_*n*_·*n*MeC_6_H_5_·1.8*n*CHCl_3_, [Cu_2_(hfacac)_4_(2)]_*n*_·*n*MeC_6_H_5_·2*n*H_2_O, [Cu_2_(hfacac)_4_(2)]_*n*_·2.8*n*C_6_H_5_Cl and [Cu_2_(hfacac)_4_(2)]_*n*_·2*n*(1,2-Cl_2_C_6_H_4_)·0.4*n*CHCl_3_·0.5*n*H_2_O

Compound	[Cu_2_(hfacac)_4_(1)]_*n*_·3.6*n*(1,2-Cl_2_C_6_H_4_)·2*n*CHCl_3_	[Zn_2_(hfacac)_4_(1)]_*n*_·*n*MeC_6_H_5_·1.8*n*CHCl_3_	[Cu_2_(hfacac)_4_(2)]_*n*_·*n*MeC_6_H_5_·2*n*H_2_O	[Cu_2_(hfacac)_4_(2)]_*n*_·2.8*n*C_6_H_5_Cl	[Cu_2_(hfacac)_4_(2)]_*n*_·2*n*(1,2-Cl_2_C_6_H_4_)·0.4*n*CHCl_3_·0.5*n*H_2_O
Empirical formula	C_95.6_H_60.4_Cl_13.2_Cu_2_F_24_N_6_O_10_	C_80.80_H_53.80_Cl_5.40_F_24_N_6_O_10_Zn_2_	C_81_H_56_Cu_2_F_24_N_6_O_12_	C_90.8_H_61_Cl_2.8_Cu_2_F_24_N_6_O_10_	C_86.40_H_56.40_Cl_5.20_Cu_2_F_24_N_6_O_10.50_
Formula weight	2504.12	2046.87	1888.39	2078.39	2113.99
Crystal system	Monoclinic	Monoclinic	Triclinic	Triclinic	Triclinic
Space group	*P*2_1_*/n*	*P*2_1_*/n*	*P*1̄	*P*1̄	*P*1̄
*a* [Å]	11.9740(4)	14.31144(10)	8.9300(2)	8.9544(2)	9.0105(3)
*b* [Å]	24.5642(6)	14.96748(10)	15.7218(3)	15.7417(4)	15.1823(4)
*c* [Å]	19.0265(6)	22.80527(13)	16.4219(3)	16.3905(4)	19.9645(3)
*α* [°]	90	90	100.0302(15)	100.053(2)	109.391(2)
*β* [°]	94.874(3)	93.1070(6)	93.7146(16)	94.071(2)	92.202(2)
*γ* [°]	90	90	96.8981(18)	97.071(2)	100.595(2)
*V* [Å^3^]	5576.1(3)	4877.85(5)	2245.22(8)	2247.46(10)	2517.67(12)
*Z*	2	2	1	1	1
*D*_c_ [g cm^−3^]	1.491	1.394	1.397	1.536	1.394
*T* [K]	130	160	160	160	160
Wavelength [Å]	1.34143	1.54184	1.54184	1.54184	1.54184
*μ* [mm^−1^]	4.547	2.854	1.554	2.342	2.676
*F*(000)	2509	2057	954	1049	1063
Crystal size [mm^3^]	0.20 × 0.14 × 0.08	0.613 × 0.384 × 0.227	0.156 × 0.085 × 0.067	0.504 × 0.178 × 0.075	0.135 × 0.08 × 0.018
Crystal description	Green plate	Colourless prism	Blue prism	Green prism	Green plate
*θ* range (data collect.) [°]	2.561 to 56.819	3.534 to 76.550	2.743 to 79.422	2.750 to 79.570	3.155 to 74.503
Index ranges	−13 ≤ *h* ≤ 14, −30 ≤ *k* ≤ 28, −23 ≤ *l* ≤ 18	−18 ≤ *h* ≤ 17, −18 ≤ *k* ≤ 18, −28 ≤ *l* ≤ 28	−10 ≤ *h* ≤ 8, −19 ≤ *k* ≤ 19, −20 ≤*l* ≤ 20	−11 ≤ *h* ≤ 10, −18 ≤ *k* ≤ 15, −19 ≤ *l* ≤ 20	−11 ≤ *h* ≤ 10, −18 ≤ *k* ≤ 18, −24 ≤ *l* ≤ 23
Measured Refl's.	62 274	81 497	29 179	33 912	39 796
Indep't Refl's	11 193	10 223	9228	8919	9979
*R*_int_	0.1234	0.0282	0.0311	0.0466	0.0629
Refl's *I* > 2*σ* (*I*)	8115	9342	8012	7542	6887
Completeness to *θ*	99.8% to 53.597°	100% to 67.684°	97.7% to 67.684°	95.1% to 67.684°	97.7% to 67.684°
Redundancy	5.56	7.97	3.16	3.80	3.99
Absorption correction	Multi-scan	Gaussian	Gaussian	Gaussian	Gaussian
Max. and min. transmission	0.560 and 0.004	1.000 and 0.122	1.000 and 0.675	1.000 and 0.286	1.000 and 0.596
Data/restraints/parameters	11 193/18/635	10 223/190/724	9228/202/731	8919/208/714	9979/571/882
Gof on *F*^2^	1.027	1.042	1.070	1.040	1.209
*R*_1_ [*I* > 2*σ* (*I*)]	0.1224	0.0797	0.0758	0.0932	0.0984
w*R*_2_ [*I* > 2*σ* (*I*)]	0.3685	0.2452	0.2299	0.2661	0.2869
*R*_1_ all data	0.1547	0.0839	0.0830	0.1035	0.1225
w*R*_2_ all data	0.4112	0.2527	0.2463	0.2839	0.3162
Largest diff. peak and hole [e Å^−3^]	1.889 and −1.099	1.235 and −0.791	1.818 and −0.627	2.229 and −1.086	1.390 and −0.363
CCDC	2162894	2162898	2162896	2162897	2162899

## Results and discussion

### Ligand synthesis and characterization

Compounds 1 and 2 were prepared using an established route for related bis(terpyridine) ligands (Scheme 4).^30^ Bouveault aldehyde syntheses^31^ starting from the reaction of 1,4-dibromo-2,5-bis(2-phenylethoxy)benzene or 1,4-dibromo-2,5-bis(3-phenylpropoxy)benzene with *n*-BuLi and DMF in dry Et_2_O at 0 °C, gave 1a (59%) and 2a (37%), respectively. Despite the use of excess of *n*-BuLi and DMF, monoaldehyde by-products persisted and purification by column chromatography was required. Finally, Hanan and Wang's one-pot strategy,^2^ provided the desired terpyridines 1 (35%) and 2 (36%) after filtration and washing with water, EtOH and Et_2_O, and no further purification was needed.

The ^1^H and ^13^C{^1^H} NMR spectra of intermediates 1a–1b and 1–2 were assigned using NOESY, COSY, HMQC and HMBC techniques (Fig. S1–S12†). The spectroscopic signatures are consistent with the structures displayed in Scheme 4. Melting point determination, ATR-IR spectroscopies (Fig. S13–S16†), UV-vis (Fig. S17†) and MALDI-TOF mass spectrometry (Fig. S18–S21†) and either HR-ESI mass spectrometry (Fig. S22–S23†) or elemental analysis complemented the characterisation (see ESI† for full details).

### Single crystal structures of 1a and 2a

**Fig. 1:**
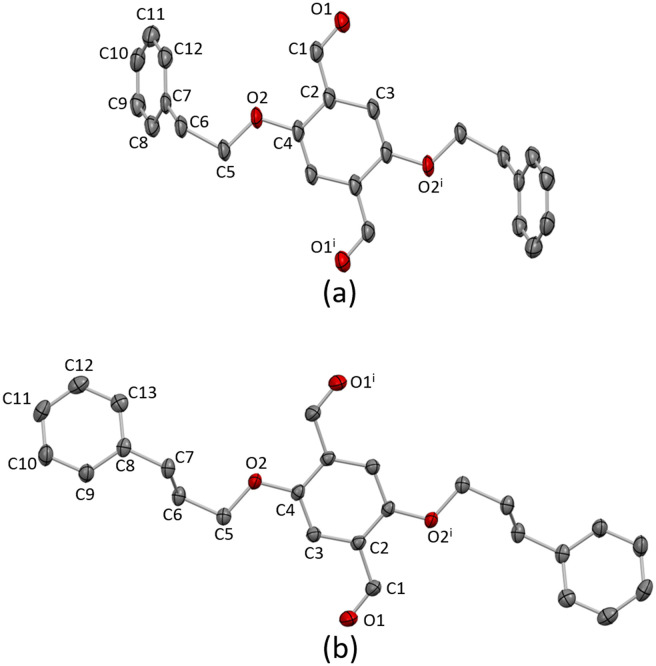
The molecular structures of (a) 1a and (b) 2a. H atoms are omitted for clarity, and thermal ellipsoids are drawn at 40% probability level. Symmetry code: i = 1 − *x*, −*y*, 1 − *z*.

Single crystals of 1a grew from the chromatographic fractions after purification (EtOAc in cyclohexane, 2–5% gradient). X-ray quality crystals of 2a were obtained as a hot EtOAc solution of 2a was allowed to cool to −20 °C. 1a crystallises in the monoclinic space group *P*2_1_/*n* and 2a in the orthorhombic *Pbca* space group. In both 1a and 2a, the asymmetric unit contains one crystallographic independent half-molecule and the second half is generated by inversion with the inversion centre located in the arene core centroid. The conformations of 1a and 2a are slightly different (Fig. 1). The extension of the phenylalkoxy substituent by the addition of an additional CH_2_ has a considerable impact on the crystal packing.

**Fig. 2:**
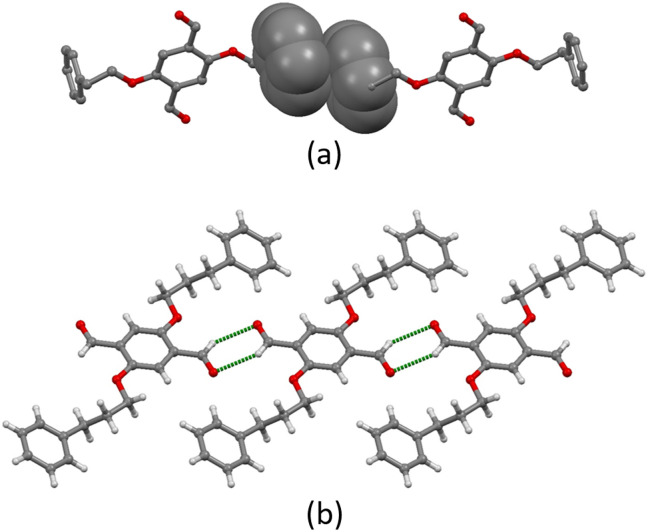
(a) Face-to-face π-stacking between two molecules of 1a. H atoms are omitted for clarity. (b) C–H⋯O hydrogen bonding between molecules of 2a leads to ribbons.

Intermolecular interactions in the crystal lattice in 1a arise from a combination of C–H⋯O hydrogen bonds, short C–H⋯π(arene) contacts, and arene–arene π-stacking. The C–H⋯O bonds arise from the oxygen atom of the aldehydes and the hydrogen atoms H5A and H5B attached to C5, with C⋯O distance of 3.12 Å (C–H⋯O range of 2.65–2.95 Å) and C–H⋯O angles range of 90.4–126.3°. Only one crystallographically independent π-stacking interaction occurs (Fig. 2a). The terminal phenyl ring containing C10 stacks with the neighbouring ring containing C10^ii^ across an inversion centre (symmetry code ii = 1 − *x*, 1 − *y*, −*z*). The rings are offset with respect to each other and the centroid⋯centroid separation is 4.12 Å. The interactions are then supplemented by short C–H⋯π(arene) contacts.

In contrast, the packing in 2a is dominated by C–H⋯O hydrogen bonds. Two CHO groups interact *via* a pair of C–H⋯O hydrogen bonds, generating a six-membered ring through a centrosymmetric arrangement; hydrogen bond metrics are C1^ii^⋯O1 = 3.27 Å (C1^ii^–H1^ii^⋯O1 = 2.52 Å), C1^ii^–H1^ii^⋯O1 = 135.9° (symmetry code ii = −*x*, −*y*, 1 − *z*). The interconnection of the CHO groups arranges the molecules into 1D ribbons as shown in Fig. 2b. The individual ribbons are parallel with respect to each other and the stacking is slightly staggered following an ABAB pattern (Fig. S24†). Weak short C–H⋯π(arene) interactions link the different stacks.

The self-association of aromatic aldehydes dimers *via* C–H⋯O interactions is rare. A search of the Cambridge Structural Database (CSD v. 2021.3.0, April 2022) for structures containing aromatic aldehydes reveals that only 157 out of 4039 crystal structures form dimers of the type found in 2a. Statistical analysis, using normalised H, reports mean distances of 2.57(10) and 2.58(9) Å for the pair of C–H⋯O interactions involved in dimer formation, with C–H⋯O angles of 130(14) and 129(14)°, respectively. In our case, a C–H⋯O distance of 2.42 Å and an angle of 133.6° (with normalised H) are consistent.

### Crystal growth experiments

Solutions of [M(hfacac)_2_]·*x*H_2_O (M = Cu, *x* = 1; M = Zn, *x* = 2) in either toluene, chlorobenzene or 1,2-dichlorobenzene were layered over a chloroform solution of 1 or 2 at room temperature. The reactions were carried out using molar metal/ligand ratios of 2 : 1 (see ESI† for full details). For each ligand, X-ray quality crystals were obtained with different solvent combinations, leading to sets of crystals that led to the five crystal structures listed in Tables 2 and 3.

**Table 3:** Crystal structures and the experimental solvent for [Cu(hfacac)_2_]·H_2_O and [Zn(hfacac)_2_]·2H_2_O, and space groups. The solvent for ligands 1 and 2 was CHCl_3_

Coordination polymer	Solvent for [M(hfacac)_2_]·*x*H_2_O	Space group
[Cu_2_(hfacac)_4_(1)]_*n*_·3.6*n*(1,2-Cl_2_C_6_H_4_)·2*n*CHCl_3_	1,2-Dichlorobenzene	*P*2_1_/*n*
[Zn_2_(hfacac)_4_(1)]_*n*_·*n*MeC_6_H_5_·1.8*n*CHCl_3_	Toluene	*P*2_1_/*n*
[Cu_2_(hfacac)_4_(2)]_*n*_·*n*MeC_6_H_5_·2*n*H_2_O	Toluene	*P*1̄
[Cu_2_(hfacac)_4_(2)]_*n*_·2.8*n*C_6_H_5_Cl	Chlorobenzene	*P*1̄
[Cu_2_(hfacac)_4_(2)]_*n*_·2*n*(1,2-Cl_2_C_6_H_4_)·0.4*n*CHCl_3_·0.5*n*H_2_O	1,2-Dichlorobenzene	*P*1̄

**Scheme 5:**
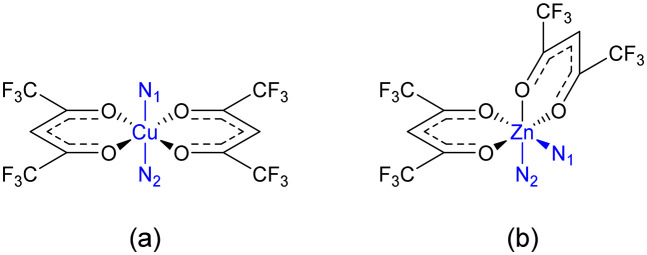
Schematic representations of (a) *trans*-{Cu(hfacac)_2_(N_1_)(N_2_)} and (b) *cis*-{Zn(hfacac)_2_(N_1_)(N_2_)} fragments. N_1_ and N_2_ originate from two different bis(tpy) ligands.

Structural analysis confirmed the assembly of a 2D-coordination polymer in each case, comprising solvated coordination networks with the general formula [M_2_(hfacac)_4_(**L**)]_*n*_ (M = Cu, Zn). From the reaction between 2 and [Cu(hfacac)_2_]·H_2_O, single crystals grew from three solvent combinations yielding [Cu_2_(hfacac)_4_(2)]_*n*_·*n*MeC_6_H_5_·2*n*H_2_O (from toluene/CHCl_3_), [Cu_2_(hfacac)_4_(2)]_*n*_·2.8*n*C_6_H_5_Cl (from chlorobenzene/CHCl_3_) and [Cu_2_(hfacac)_4_(2)]_*n*_·2*n*(1,2-Cl_2_C_6_H_4_)·0.4*n*CHCl_3_·0.5*n*H_2_O (from 1,2-dichlorobenzene/CHCl_3_). The first two are isostructural networks which crystallise in the triclinic space group *P*1̄ and possess comparable cell dimensions (*a* = 8.9300(2), *b* = 15.7218(3), *c* = 16.4219(3) Å, *α* = 100.0302(15), *β* = 93.7146(16), *γ* = 96.8981(18)° for [Cu_2_(hfacac)_4_(2)]_*n*_·*n*MeC_6_H_5_·2*n*H_2_O, and *a* = 8.9544(2), *b* = 15.7417(4), *c* = 16.3905(4) Å, *α* = 100.053(2), *β* = 94.071(2), *γ* = 97.071(2)° for [Cu_2_(hfacac)_4_(2)]_*n*_·2.8*n*C_6_H_5_Cl). Therefore, we only discuss in detail the structure of [Cu_2_(hfacac)_4_(2)]_*n*_·2.8*n*C_6_H_5_Cl. In [Cu_2_(hfacac)_4_(2)]_*n*_·2*n*(1,2-Cl_2_C_6_H_4_)·0.4*n*CHCl_3_·0.5*n*H_2_O, a change in conformation of the 3,2′:6′,3′′-tpy domains justifies a separate description of this structure. All five assemblies are (4,4)-nets, but two structurally distinct designs can be identified and are discussed separately: (4,4)-nets with a *trans*-arrangement of the {Cu(hfacac)_2_(N_1_)(N_2_)} units and a (4,4)-net with *cis*-arrangement of the {Zn(hfacac)_2_(N_1_)(N_2_)} units (Scheme 5).

### (4,4)-Networks containing Cu(hfacac)_2_

[Cu_2_(hfacac)_4_(1)]_*n*_·3.6*n*(1,2-Cl_2_C_6_H_4_)·2*n*CHCl_3_ crystallises in the monoclinic space group *P*2_1_/*n*, while [Cu_2_(hfacac)_4_(2)]_*n*_·2.8*n*C_6_H_5_Cl and [Cu_2_(hfacac)_4_(2)]_*n*_·2*n*(1,2-Cl_2_C_6_H_4_)·0.4*n*CHCl_3_·0.5*n*H_2_O crystallise in the triclinic space group *P*1̄. In [Cu_2_(hfacac)_4_(1)]_*n*_·3.6*n*(1,2-Cl_2_C_6_H_4_)·2*n*CHCl_3_ the asymmetric unit contains one independent copper atom and one independent half-ligand (Fig. S25†). In contrast, the asymmetric units of [Cu_2_(hfacac)_4_(2)]_*n*_·2.8*n*C_6_H_5_Cl and [Cu_2_(hfacac)_4_(2)]_*n*_·2*n*(1,2-Cl_2_C_6_H_4_)·0.4*n*CHCl_3_·0.5*n*H_2_O contain two independent half copper atoms and one independent half-ligand (Fig. S26–S27†). The copper centres lie on crystallographic inversion centres. For [Cu_2_(hfacac)_4_(2)]_*n*_·2*n*(1,2-Cl_2_C_6_H_4_)·0.4*n*CHCl_3_·0.5*n*H_2_O, Cu2 is disordered over an inversion centre (Fig. S27†); for the discussion of the polymer, only the mean position is considered. In all three compounds, each Cu(ii) centre is octahedrally coordinated with a *trans*-arrangement of coordinated [hfacac]^−^ ligands. Bond lengths and angles in the Cu(ii) coordination sphere are typical, with Cu–N distances in the range 1.981(5)–2.048(9) Å and Cu–O in the range 1.979(4)–2.330(5) Å. In both coordinated ligand 1 and 2, the two tpy domains are crystallographically related, with the phenylene spacer centroid lying on an inversion centre. Each bis(tpy) ligand binds through the outer nitrogen donors to four Cu(ii) centers, therefore acting as the 4-connecting node and directing the assembly of a (4,4)-net. The tpy units are non-planar and the torsion angles between the ring planes range between 4.5° and 33.6° (Table 4). Torsion angles of 34.5°, 39.1° and 51.1° between the planes of the central pyridine ring and the central arene spacer are typical for minimising H⋯H inter-ring repulsions.

**Table 4:** Angles between the planes of pairs of connected rings in the coordinated ligands

Compound	py–py/°	py_N2_-phenyl^a^/°
[Cu_2_(hfacac)_4_(1)]_*n*_·3.6*n*(1,2-Cl_2_C_6_H_4_)·2*n*CHCl_3_	27.5, 33.6	39.1
[Cu_2_(hfacac)_4_(2)]_*n*_·2.8*n*C_6_H_5_Cl	4.5, 32.9	34.5
[Cu_2_(hfacac)_4_(2)]_*n*_·2*n*(1,2-Cl_2_C_6_H_4_)·0.4*n*CHCl_3_·0.5*n*H_2_O	5.9, 28.4	51.1

a N2, for labelling schemes see Fig. S25–S27.†

**Fig. 3:**
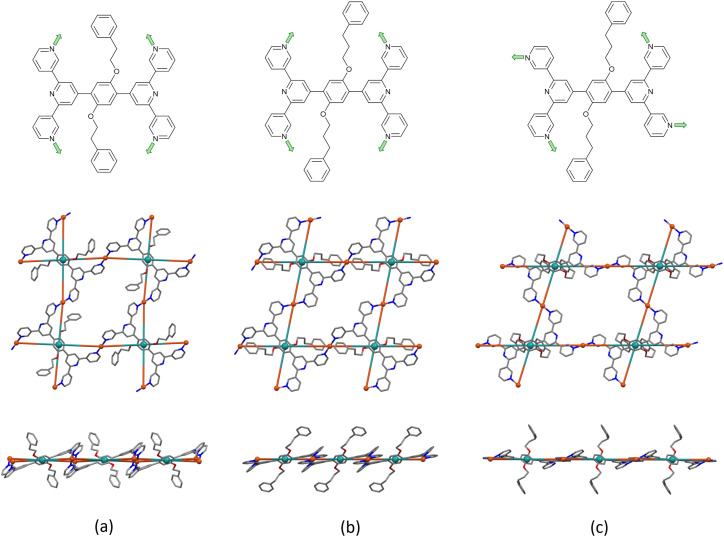
Comparison of the ligand conformations and the (4,4) nets in (a) [Cu_2_(hfacac)_4_(1)]_*n*_·3.6*n*(1,2-Cl_2_C_6_H_4_)·2*n*CHCl_3_, (b) [Cu_2_(hfacac)_4_(2)]_*n*_·2.8*n*C_6_H_5_Cl, and (c) [Cu_2_(hfacac)_4_(2)]_*n*_·2*n*(1,2-Cl_2_C_6_H_4_)·0.4*n*CHCl_3_·0.5*n*H_2_O. Top, conformation of the coordinated ligand; middle, looking down on the sheets; bottom, (4,4) nets observed through the mean plane determined by the nodes of the ligand centroids (green). For clarity, H atoms, coordinated [hfacac]^−^ ligands and solvent are omitted, and only major occupancies are shown.

In [Cu_2_(hfacac)_4_(1)]_*n*_·3.6*n*(1,2-Cl_2_C_6_H_4_)·2*n*CHCl_3_ and [Cu_2_(hfacac)_4_(2)]_*n*_·2.8*n*C_6_H_5_Cl both ligand 1 and 2 adopt conformation **B**. Interestingly, with ligand 2, a solvent change from chlorobenzene (or toluene) to 1,2-dichlorobenzene leads to a conformational change of the tpy groups within the 2D-polymer (Fig. 3b and c, top). In fact, in [Cu_2_(hfacac)_4_(2)]_*n*_·2*n*(1,2-Cl_2_C_6_H_4_)·0.4*n*CHCl_3_·0.5*n*H_2_O, ligand 2 displays conformation **C**, although this does not lead to a significant change in the network (Fig. 3b and c, middle). In all three structures, the combination of ligand 1 or 2 with Cu(hfacac)_2_ leads to a 2D-net directed by the tetratopic ligands. The centroids of the phenylene spacers are the nodes of the network, whereas the copper centres act as linkers (Fig. 3, middle). The network in [Cu_2_(hfacac)_4_(1)]_*n*_·3.6*n*(1,2-Cl_2_C_6_H_4_)·2*n*CHCl_3_ contains a rhombic shortest circuit with internal angles of 87.0 and 93.0° and a node⋯node distance of 16.94 Å. The copper atoms are close to the plane of the (4,4)-net generating a small deformation in the structure (Fig. 3a, middle and bottom). In contrast, in [Cu_2_(hfacac)_4_(2)]_*n*_·2.8*n*C_6_H_5_Cl and [Cu_2_(hfacac)_4_(2)]_*n*_·2*n*(1,2-Cl_2_C_6_H_4_)·0.4*n*CHCl_3_·0.5*n*H_2_O, the shortest circuits are parallelograms (Fig. 3b and c, middle) with the copper centres lying in the plane (Fig. 3b and c, bottom). The conformational change of ligand 2 does not appear to play a crucial role in the assembly. Indeed, from [Cu_2_(hfacac)_4_(2)]_*n*_·2.8*n*C_6_H_5_Cl to [Cu_2_(hfacac)_4_(2)]_*n*_·2*n*(1,2-Cl_2_C_6_H_4_)·0.4*n*CHCl_3_·0.5*n*H_2_O, only minor changes occur in the distances and angles between the individual nodes (Table 5).

**Table 5:** Distances and angles between the nodes in the (4,4)-nets

Compound	Node–node distance/Å	Internal angles of rhombus in (4,4) net/°
[Cu_2_(hfacac)_4_(1)]_*n*_·3.6*n*(1,2-Cl_2_C_6_H_4_)·2*n*CHCl_3_	16.94	87.0, 93.0
[Cu_2_(hfacac)_4_(2)]_*n*_·2.8*n*C_6_H_5_Cl	16.42, 17.12	78.8, 101.2
[Cu_2_(hfacac)_4_(2)]_*n*_·2*n*(1,2-Cl_2_C_6_H_4_)·0.4*n*CHCl_3_·0.5*n*H_2_O	16.17, 20.68	73.0, 107.0

**Fig. 4:**
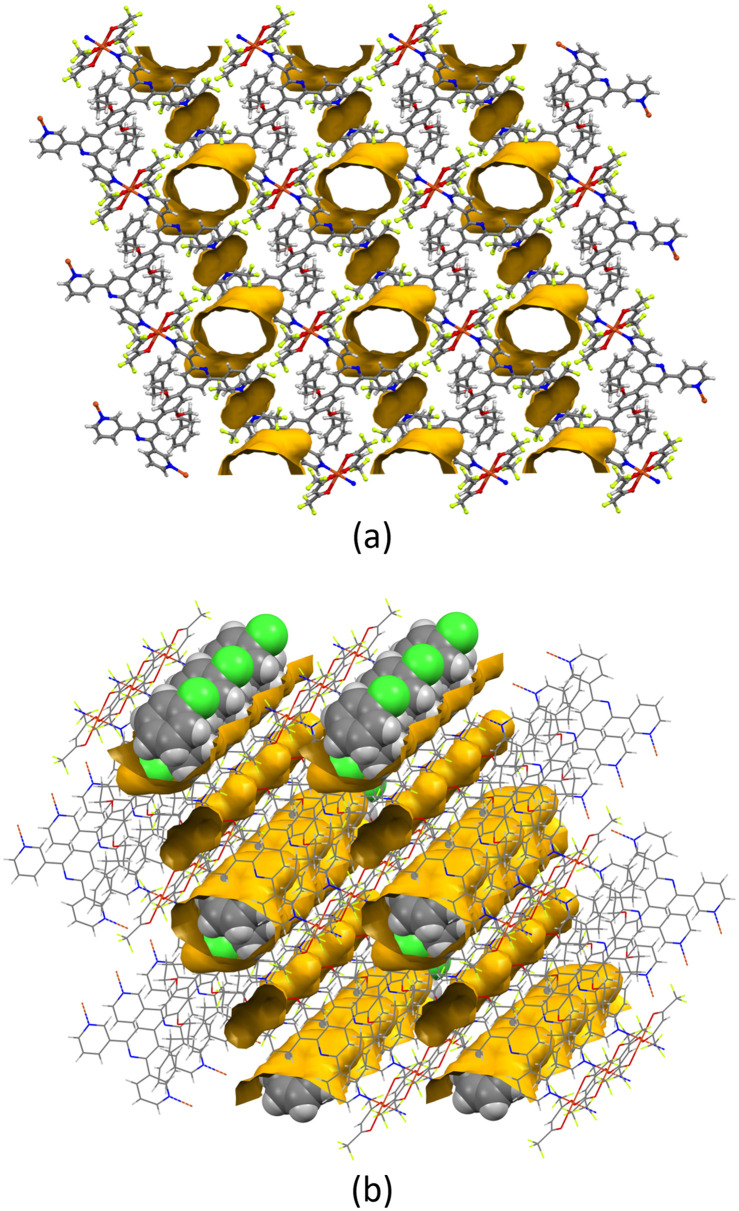
(a) Illustration of the void space in the crystal lattices of [Cu_2_(hfacac)_4_(2)]_*n*_·2.8*n*C_6_H_5_Cl (*ca.* 21% void) with channels following the *a*-axis. (b) Representation of the same structure with solvent molecules. The void was calculated from the structure without solvent molecules. Subsequently, the same structure with solvent molecules was superimposed on the one without, revealing the chlorobenzene in the channels. Contact surface map with probe radius = 1.2 Å. Calculations made with Mercury (v. 2021.3.0).^32^

All the structures considered in this section have phenylalkoxy groups pointing above and below the plane (Fig. 3, bottom). [Cu_2_(hfacac)_4_(1)]_*n*_·3.6*n*(1,2-Cl_2_C_6_H_4_)·2*n*CHCl_3_ and [Cu_2_(hfacac)_4_(2)]_*n*_·2*n*(1,2-Cl_2_C_6_H_4_)·0.4*n*CHCl_3_·0.5*n*H_2_O were crystallised in the same solvent mixture (from 1,2-dichlorobenzene/CHCl_3_) and with the same reagent concentrations. The only difference is in the length of the phenylalkoxy substituent, ligand 1 possesses a 2-phenylethoxy whereas ligand 2 possesses a 3-phenylpropoxy. Despite the difference of only one CH_2_ group, the network is significantly affected going from [Cu_2_(hfacac)_4_(1)]_*n*_·3.6*n*(1,2-Cl_2_C_6_H_4_)·2*n*CHCl_3_ to [Cu_2_(hfacac)_4_(2)]_*n*_·2*n*(1,2-Cl_2_C_6_H_4_)·0.4*n*CHCl_3_·0.5*n*H_2_O (Fig. 3a and c). It is important to note that, in [Cu_2_(hfacac)_4_(1)]_*n*_·3.6*n*(1,2-Cl_2_C_6_H_4_)·2*n*CHCl_3_, the peripheral phenyl ring is not involved in any significant interaction, whereas in [Cu_2_(hfacac)_4_(2)]_*n*_·2*n*(1,2-Cl_2_C_6_H_4_)·0.4*n*CHCl_3_·0.5*n*H_2_O, short C–H⋯π(arene) interactions link the pendant phenyl ring with the 1,2-dichlorobenzene molecule and the phenylene spacer of the ligand in the adjacent sheet (Fig. S28†). The 3-phenylpropoxy substituent also engages in weak interactions in the crystal structure of [Cu_2_(hfacac)_4_(2)]_*n*_·2.8*n*C_6_H_5_Cl, where the terminal phenyl ring stacks with the central arene spacer of the ligand 2 contained in a neighbouring (4,4)-net (Fig. S29†). In each lattice, the individual layers pack with the cavities running down the crystallographic *a*-axis (Fig. 4). The solvent molecules are accommodated within these open channels.

### (4,4)-Network containing Zn(hfacac)_2_

**Scheme 6:**
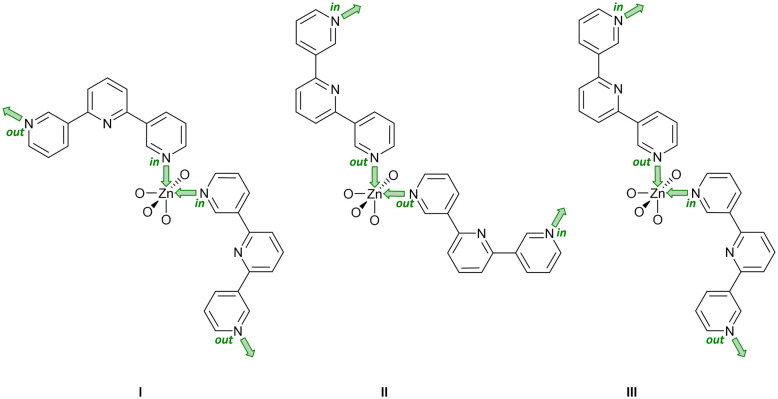
With pairs of 3,2′:6′,3′′-tpy ligands in conformation **C** (Scheme 1), there are three possible coordination topology (I)–(III) at a Zn atom that does not lie on an inversion center. The labels *in* and *out* indicate the orientation of the lone pair of each coordinating N atom relative to the central N atom of the 3,2′:6′,3′′-tpy unit. Only limiting planar conformations are shown and coordinated oxygen atoms arise from [hfacac]^−^ ligands.

Single crystals of [Zn_2_(hfacac)_4_(1)]_*n*_·*n*MeC_6_H_5_·1.8*n*CHCl_3_ were grown by layering a toluene solution of [Zn(hfacac)_2_]·2H_2_O over a CHCl_3_ solution of 1. [Zn_2_(hfacac)_4_(1)]_*n*_·*n*MeC_6_H_5_·1.8*n*CHCl_3_ crystallises in the monoclinic space group *P*2_1_/*n.* The asymmetric unit contains one independent Zn(ii) centre and one independent half-ligand; the second half is generated by inversion (Fig. S30†). The Zn atom is octahedrally sited and coordinated to four oxygen atoms of two chelating [hfacac]^−^ ligands (Zn–O in the range 2.083(2)–2.136(2) Å), and to two pyridine donor atoms of two different ligands 1 (Zn–N = 2.100(1), 2.120(3) Å), which are in a *cis*-arrangement. The coordination sphere is distorted with an N–Zn–N bond angle of 97.70(10)° and similar values have been reported previously for *cis*-{Zn(hfacac)_2_(N_1_)(N_2_)} complexes with substituted pyridine donors.^33–35^ The tpy domain adopts conformation **C** (Scheme 1) and the angles between the planes of the rings containing N1/N2 and N2/N3 are 25.2 and 3.9°, respectively. The phenylene spacer is rotated 50.7° relative to the pyridine ring containing N2. Note that when the metal is not on an inversion centre and the two 3,2′:6′,3′′-tpy units are in conformation **C** (Scheme 1), three spatial arrangements of tpy domains are possible (Scheme 6). In [Zn_2_(hfacac)_4_(1)]_*n*_·*n*MeC_6_H_5_·1.8*n*CHCl_3_, pairs of ligand 1 are arranged in the orientation III as shown in Scheme 6.

**Fig. 5:**
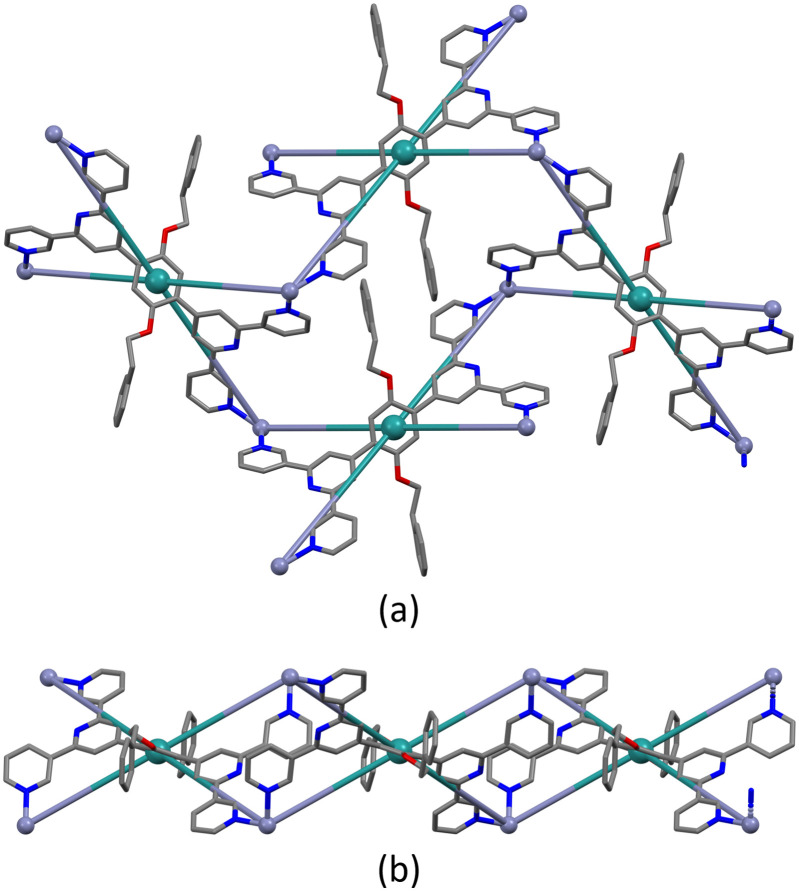
Shortest circuit with four nodes (ligand centroids, green) of the (4,4) net in [Zn_2_(hfacac)_4_(1)]_*n*_·*n*MeC_6_H_5_·1.8*n*CHCl_3_; (a) looking down on the sheet, (b) (4,4) net observed through the mean plane determined by the nodes. For clarity, H atoms, coordinated [hfacac]^−^ ligands and solvent are omitted, and only major occupancies are shown.

As with the complexes described in the previous section, ligand 1 behaves as a 4-connecting node, coordinating four different Zn(ii) ions and directing the assembly of a planar (4,4)-net (Fig. 5a). The distance between adjacent ligand nodes (centroids of the phenylene spacers) is 15.11 Å and the internal angles of the rhombic shortest circuit are 59.4 and 120.6°. Compared to the copper(ii) structures reported in this work, the topology of the zinc(ii) network is identical. However, structural differences can be seen by examination of how the metal linkers and the phenylalkoxy tails are arranged. The zinc(ii) centres are disposed alternately above and beneath the plane generated by the ligand nodes, while the 2-phenylethoxy chains are located in the plane (Fig. 5b). Each cavity of the network accommodates two 2-phenylethoxy tails, both originating from the same individual 2D-coordination polymer, and the pendant phenyl rings interact *via* face-to-face π-interaction across an inversion centre (Fig. S31†).

**Fig. 6:**
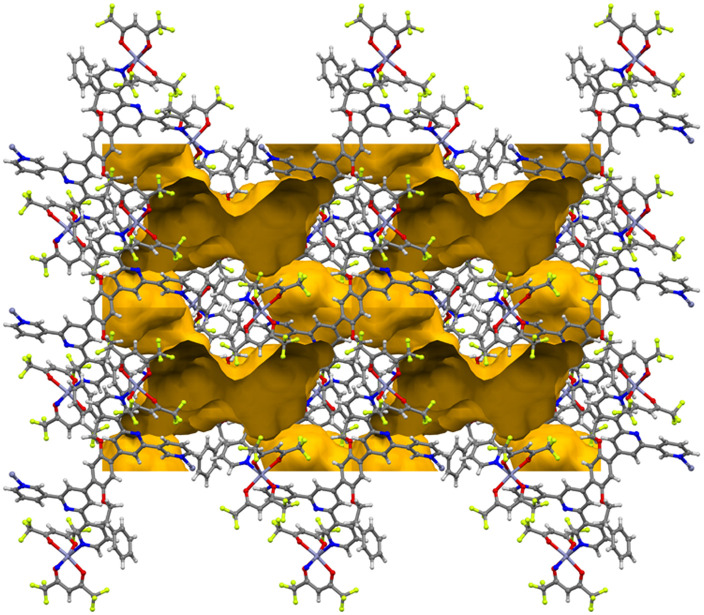
Illustration of the void space in the crystal lattices of [Zn_2_(hfacac)_4_(1)]_*n*_·*n*MeC_6_H_5_·1.8*n*CHCl_3_ (*ca.* 33% void) following the *a*-axis. Contact surface map with probe radius = 1.2 Å. Calculations made with Mercury (v. 2021.3.0).^32^

Note that a change from a *trans* to a *cis* arrangement of the N-donors on going from the Cu(ii) to Zn(ii) structures modifies the architecture of the assembly without changing the underlying (4,4)-network. On the other hand, we have recently reported a planar (4,4)-net with *trans*-{Cu(hfacac)_2_(N_1_)(N_2_)} domains lying above and below the plane displaying a similar arrangement of metal linkers.^29^ In [Zn_2_(hfacac)_4_(1)]_*n*_·*n*MeC_6_H_5_·1.8*n*CHCl_3_, viewed down the crystallographic *b*-axis, the individual layers are parallel to each other (Fig. S32†). The packing of the sheets shows a zig-zag arrangement with the CF_3_ groups protruding out of the plane. Interactions between the layers are dominated by short C–H⋯F–C contacts between 2-phenylethoxy substituents in one sheet and the CF_3_ groups from the neighbouring one. However, since the CF_3_ groups are disordered, a detailed discussion is not meaningful. Removal of the solvent molecules from the structure reveals cavities (Fig. 6) rather than open channels in which they are located.

### Bulk sample analysis

**Fig. 7:**
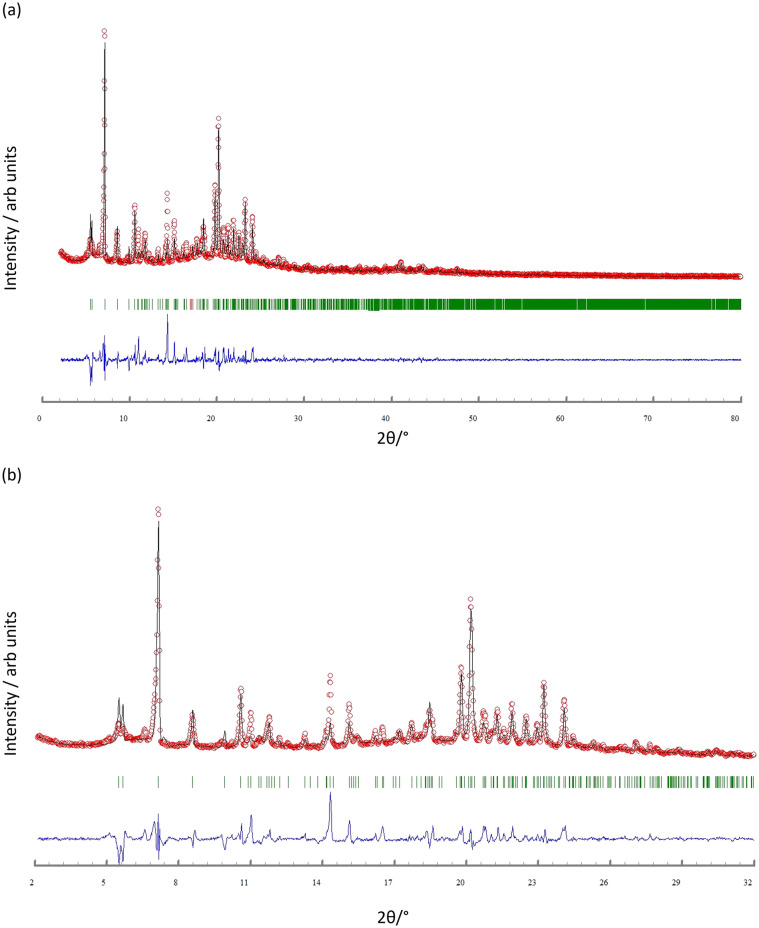
(a) X-ray diffraction (CuKα_1_ radiation) pattern (red circles) of the bulk crystalline material of [Cu_2_(hfacac)_4_(2)]_*n*_·*n*MeC_6_H_5_·2*n*H_2_O, fitting the predicted pattern from the single crystal structure. The black lines are the best fit from Rietveld refinements, and green lines display the Bragg peak positions. The blue plot gives the difference between calculated and experimental points. (b) Expansion in the 2–32° range.

Once single crystals had been selected for single-crystal X-ray structure diffraction, the bulk materials were analysed by powder X-ray diffraction (PXRD). All the crystalline materials were sensitive to loss of solvent upon exposure to air and were thus measured wet and without washing them. For [Cu_2_(hfacac)_4_(2)]_*n*_·*n*MeC_6_H_5_·2*n*H_2_O, all peaks in the experimental PXRD pattern (red traces in Fig. 7) correspond to the predicted pattern from the single crystal structure (black traces in Fig. 7). Preferred orientations of the crystallites explain the differences in the relative intensities of the peaks (blue traces in Fig. 7). In the same manner, for [Zn_2_(hfacac)_4_(1)]_*n*_·*n*MeC_6_H_5_·1.8*n*CHCl_3_, a good match was found between the experimental PXRD pattern for the bulk material and the pattern predicted from the single-crystal structure (Fig. S33†). On the other hand, the fitting between the calculated and experimental patterns of the remaining compounds did not show a good match and are shown in Fig. S34–S36.† Unlike the single-crystal X-ray determinations, which were carried out at low temperatures, the PXRD patterns were recorded at room temperature. Phase transition and solvent loss can affect the PXRD significantly and in view of the observed facile solvent loss, we did not investigate the crystallographic properties of the bulk material further.

The solid state IR spectra of the dried copper(ii) coordination polymers are shown in Fig. S37–S40.† Not surprisingly, given the similarity of the structures, the absorption of the 1,3-diketonate and the fingerprint regions are almost identical.

## Conclusions

We have synthesised and characterised two bis(3,2′:6′,3′-tpy) 1 and 2 ligands, featuring 2-phenylethoxy and 3-phenylpropoxy substituents attached to the 1,4-phenylene spacer, respectively. Ligands 1 and 2 were then allowed to react with [M(hfacac)_2_]·*x*H_2_O (M = Cu, *x* = 1; M = Zn, *x* = 2) under ambient condition of crystal growth in a combination of CHCl_3_ and an aromatic solvent. The single crystal determination revealed the formation of the 2D-coordination networks [Cu_2_(hfacac)_4_(1)]_*n*_·3.6*n*(1,2-Cl_2_C_6_H_4_)·2*n*CHCl_3_, [Zn_2_(hfacac)_4_(1)]_*n*_·*n*MeC_6_H_5_·1.8*n*CHCl_3_, [Cu_2_(hfacac)_4_(2)]_*n*_·*n*MeC_6_H_5_·2*n*H_2_O, [Cu_2_(hfacac)_4_(2)]_*n*_·2.8*n*C_6_H_5_Cl and [Cu_2_(hfacac)_4_(2)]_*n*_·2*n*(1,2-Cl_2_C_6_H_4_)·0.4*n*CHCl_3_·0.5*n*H_2_O. [Cu_2_(hfacac)_4_(2)]_*n*_·*n*MeC_6_H_5_·2*n*H_2_O and [Cu_2_(hfacac)_4_(2)]_*n*_·2.8*n*C_6_H_5_Cl are isostructural. In all assemblies, the bis(3,2′:6′,3′′-tpy) ligands coordinate four [M(hfacac)_2_] (M = Cu; M = Zn) units directing the formation of planar (4,4)-nets, with the centroids of the phenylene spacers of 1 or 2 acting as nodes and the metal ions working as linkers.

In the copper(ii) coordination polymers the metal ions display a *trans*-arrangement of the N-donor atoms. Differences, such as phenylalkoxy chain length or conformational changes in the 3,2′:6′,3′′-tpy groups, do not change significantly the motif and distinct features remain identical within the series. The Cu(ii) centres are located near or in the plane (determined by the nodes) and the phenylalkoxy chains are directed outwards from the individual sheets. By contrast, in [Zn_2_(hfacac)_4_(1)]_*n*_·*n*MeC_6_H_5_·1.8*n*CHCl_3_, the Zn(ii) centres have a *cis*-arrangement of the N atoms and are arranged alternately above and below the network. Pairs of 2-phenylethoxy tails are lodged in each cavity of the (4,4)-net interacting with each other *via* face-to-face π-interaction.

This work showed that the assembly of planar (4,4)-nets by combining ligands 1 or 2 with [M(hfacac)_2_]·*x*H_2_O (M = Cu, *x* = 1; M = Zn, *x* = 2) is independent upon the choice of the crystallization solvents. A switch from Cu(ii) to Zn(ii) influences the orientation of the metal linkers but does not change the topology of the network.

## Author contributions

Methodology and data analysis: G. M.; crystallography: A. P. and B. S.; PXRD: G. M.; supervision, project administration and funding acquisition: E. C. C. and C. E. H.; writing—original draft, G. M.; writing—review and editing: all authors. All authors have read and agreed to the published version of the manuscript.

## Conflicts of interest

There are no conflicts to declare.

## Supplementary Material

CE-024-D2CE01130A-s001

CE-024-D2CE01130A-s002
