# Supplementary material for: Multitopic 3,2′:6′,3′′-terpyridine ligands as 4-connecting nodes in two-dimensional 4,4-networks
Source: CrystEngComm. 2022 Sep 23;24(40):7073–82. doi: 10.1039/d2ce01130a (PMC9575388; doi:10.1039/d2ce01130a)
Supplement: CE-024-D2CE01130A-s001 [file CE-024-D2CE01130A-s001.pdf]

Electronic Supplementary Material (ESI) for CrystEngComm.
This journal is © The Royal Society of Chemistry 2022

Supplementary Information

Multitopic 3,2':6',3''-terpyridine ligands as 4-connecting nodes in two-dimensional 4,4-networks

Giacomo Manfroni,[a] Bernhard Spingler,[b] Alessandro Prescimone,[a] Edwin C. Constable[a] and Catherine E. Housecroft*[a]

a. Department of Chemistry, University of Basel, Mattenstrasse 24a, BPR 1096, 4058-Basel, Switzerland.

b. Department of Chemistry, University of Zurich, Winterthurerstr. 190, 8057-Zurich, Switzerland.

Experimental Section

Materials and instrumentation

3-Acetylpyridine was purchased from Acros Organics and [Cu(hfacac)$_2$]·H_2O was bought from abcr GmbH. [Zn(hfacac)$_2$]·2H_2O was purchased from Sigma-Aldrich. All chemicals were used as received. 1,4-Dibromo-2,5-bis(2-phenylethoxy)benzene and 1,4-dibromo-2,5-bis(3-phenylpropoxy)benzene were prepared as previously described in literature.[1]

Analytical thin-layer chromatography was conducted with pre-coated silica gel 60 F_{254} aluminium sheets (Merck KGaA) and visualized using ultraviolet light (254 nm and 366 nm). Flash column chromatography was performed on a Biotage Selekt system with pre-packed silica gel columns (50 g Biotage® Sfär Silica HC D – High Capacity Duo 20 µm, Biotage) using ethyl acetate in cyclohexane (gradient, see below) as eluent and monitoring and collecting at 366 nm.

^{1}H and ^{13}C{^{1}H} NMR spectra were recorded on a Bruker Avance III-500 spectrometer at 298 K. The ^{1}H and ^{13}C NMR chemical shifts were referenced with respect to the residual solvent peak (δ 2.50 for DMSO-d_5 and δ 39.5 for DMSO-d_6). MALDI-TOF mass spectra were recorded on a Shimadzu MALDI 8020 using α-cyano-4-hydroxycinnamic acid (CHCA) or 2,5-dihydroxybenzoic acid (DHB) as the matrix. PerkinElmer UATR Two and Shimadzu UV-2600 instruments were used to record FT-infrared (IR) and UV-Vis absorption spectra, respectively. Melting temperatures were determined using a Stuart melting point SMP 30 device. High-resolution electrospray (HR-ESI) mass spectra were performed using a Bruker maXis 4G QTOF instrument.

Synthesis

2,5-Bis(2-phenylethoxy)terephthalaldehyde (1a)

n-BuLi in *n*-hexane (1.6 M, 3.68 mL, 5.88 mmol) was added slowly at 0 °C under N_2 atmosphere over a period of 15 min to a suspension of 1,4-dibromo-2,5-bis(2-phenylethoxy)benzene (800 mg, 1.68 mmol) in dry Et_2O (50 mL). After 4.5 h, dry DMF (0.46 mL, 5.9 mmol) was added. The resulting suspension was then stirred for a further 22 h under an N_2 atmosphere while warming up from 0 °C to room temperature. The light yellow suspension was neutralized with saturated aqueous NH_4Cl and extracted with $CHCl_3$ (3 x 75 mL). The organic layers were dried over $MgSO_4$ and concentrated *in vacuo*. The crude product was purified by column chromatography using EtOAc in cyclohexane (2-5% gradient) as eluent. Compound **1a** was isolated as a yellow microcrystalline solid (373 mg, 0.996 mmol, 59.3%). M.p. = 168.8-169.2 °C. ^{1}H NMR (500 MHz, DMSO-d_6) δ/ppm 10.25 (s, 2H, H^{CHO}), 7.40 (s, 2H, H^{A3}), 7.34-7.28 (m, 8H, H^{B2+B3}), 7.22 (m, 2H, H^{B4}), 4.36 (t, *J* = 6.5 Hz, 4H, H^a), 3.08 (t, *J* = 6.5 Hz, 4H, H^b). ^{13}C{1H} NMR (126

MHz, DMSO-d_6) δ/ppm 188.9 (C^{CHO}), 154.5 (C^{A2}), 138.3 (C^{B1}), 129.0 (C^{A1+B2}), 128.3 (C^{B3}), 126.3 (C^{B4}), 112.2 (C^{A3}), 69.7 (C^{a}), 34.8 (C^{b}). UV-Vis ($CHCl_3$, 3.0×10^{-5} mol dm^{-3}) λ/nm 274 (ε/dm^3 mol^{-1} cm^{-1} 15,640), 283 sh (12,940), 398 (6,060). MALDI-TOF-MS (matrix: DHB) m/z 374.67 $[M]^+$ (calc. 374.15). Found C 76.01, H 6.06; required for $C_{24}H_{22}O_4$: C 76.99, H 5.92.

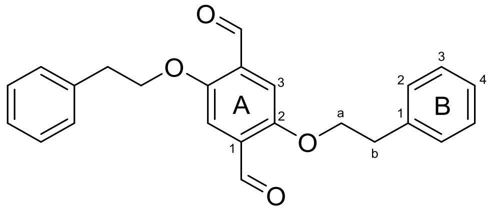

2,5-Bis(3-phenylpropoxy)terephthalaldehyde (2a)

n-BuLi in *n*-hexane (1.6 M, 7.43 mL, 11.9 mmol) was added slowly at 0 °C under N_2 atmosphere over a period of 15 min to a suspension of 1,4-dibromo-2,5-bis(3-phenylpropoxy)benzene (1.50 g, 2.97 mmol). After 2.5 h, dry DMF (0.92 mL, 12 mmol) was added. The resulting solution was then stirred for a further 22 h under an N_2 atmosphere while warming up from 0 °C to room temperature. The light yellow suspension was neutralized with saturated aqueous NH_4Cl and extracted with $CHCl_3$ (3 x 75 mL). The organic layers were dried over $MgSO_4$ and concentrated *in vacuo*. The crude material was purified by column chromatography using 5% EtOAc in cyclohexane as eluent. The solid product was recrystallized from hot EtOAc (cooled to –20 °C). Yellow crystals were isolated by filtration, washed with cold EtOAc and cyclohexane, and dried *in vacuo* for 2 h to afford **2a** (445 mg, 1.11 mmol, 37.2%). M.p. = 112.0-112.8 °C. ^{1}H NMR (500 MHz, DMSO-d_6) δ/ppm 10.34 (s, 2H, H^{CHO}), 7.38 (s, 2H, H^{A3}), 7.30-7.22 (m, 8H, H^{B2+B3}), 7.18 (m, 2H, H^{B4}), 4.14 (t, *J* = 6.2 Hz, 4H, H^{a}), 2.78 (dd, *J* = 8.5, 6.7 Hz, 4H, H^{c}), 2.12-2.04 (m, 4H, H^{b}). ^{13}C{1H} NMR (126 MHz, DMSO-d_6) δ/ppm 189.1 (C^{CHO}), 154.5 (C^{A2}), 141.4 (C^{B1}), 128.9 (C^{A1}), 128.3 (C^{B2+B3}), 125.83 (C^{B4}), 111.9 (C^{A3}), 68.3 (C^{a}), 31.6 (C^{c}), 30.2 (C^{b}). UV-Vis ($CHCl_3$, 3.0×10^{-5} mol dm^{-3}) λ/nm 276 (ε/dm^3 mol^{-1} cm^{-1} 16,060), 283 sh (13,280), 401 (6,300). MALDI-TOF-MS (matrix: CHCA) m/z 402.67 $[M]^+$ (calc. 402.18). Found C 77.41, H 6.55; required for $C_{26}H_{26}O_4$: C 77.59, H 6.51.

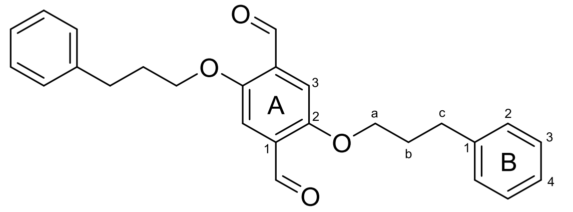

1,4-Bis(2-phenylethoxy)-2,5-bis(3,2':6',3''-terpyridin-4'-yl)benzene (1)

2,5-Bis(2-phenylethoxy)terephthalaldehyde (286 mg, 0.764 mmol) was dissolved in EtOH (20 mL), and 3-acetylpyridine (463 mg, 0.421 mL, 3.82 mmol) was added to the solution and crushed KOH (214 mg, 3.82 mmol) was added in one portion. Aqueous NH_3 solution (32%, 4.78 mL) was added slowly and the reaction mixture was stirred at room temperature for 6 days. The brownish solid that formed was collected by filtration, washed with water (2 x 20 mL), EtOH (2 x 10 mL) and Et_2O (2 x 20 mL), and then dried *in vacuo* overnight (ca. 16 h). The product was isolated as a white powder **1**, 206 mg, 0.264 mmol, 34.5%). M.p. = 248.8-249.7 °C. ^{1}H NMR (500 MHz, DMSO-d_6): δ/ppm 9.43 (dd, *J* = 2.3, 0.9 Hz, 4H, H^{A2}), 8.71 (dd, *J* = 4.7, 1.6 Hz, 4H, H^{A6}, 8.58 (ddd, *J* = 8.0, 2.3, 1.7 Hz, 4H, H^{A4}), 8.19 (s, 4H, H^{B3}), 7.60 (ddd, *J* = 8.0, 4.8, 0.9 Hz, 4H, H^{A5}), 7.48 (s, 2H, H^{C3}), 7.16 – 7.11 (m, 4H, H^{D2}), 7.09 – 7.03 (m, 6H, H^{D3+D4}), 4.41 (t,

J = 6.4 Hz, 4H, H^{a}), 2.99 (t, J = 6.4 Hz, 4H, H^{b}). $^{13}C\{1H\}$ NMR (126 MHz, DMSO-d_6) δ/ppm 153.9 (C^{B2}), 150.1 (C^{A6}), 149.9 (C^{C2}), 148.2 (C^{A2}), 147.7 (C^{C1}), 138.4 (C^{D1}), 134.4 (C^{A4}), 134.1 (C^{A3}), 128.7 (C^{D2}), 128.4 (C^{B4}), 128.1 (C^{D3}), 126.1 (C^{D4}), 123.9 (C^{A5}), 120.5 (C^{B3}), 115.6 (C^{C3}), 69.6 (C^{a}), 35.0 (C^{b}). UV-Vis ($CHCl_3$, 2.0 × 10^{-5} mol dm^{-3}) λ/nm 280 sh (ε/dm^3 mol^{-1} cm^{-1} 49,380), 315 (23,880). MALDI-TOF-MS (matrix: CHCA) m/z 781.24 $[M+H]^+$ (calc. 781.33). HR-ESI MS m/z 781.3277 $[M+H]^+$ (calc. 781.3286), 391.1680 $[M+2H]^{2+}$ (calc. 391.1679). Satisfactory elemental analysis could not be obtained.

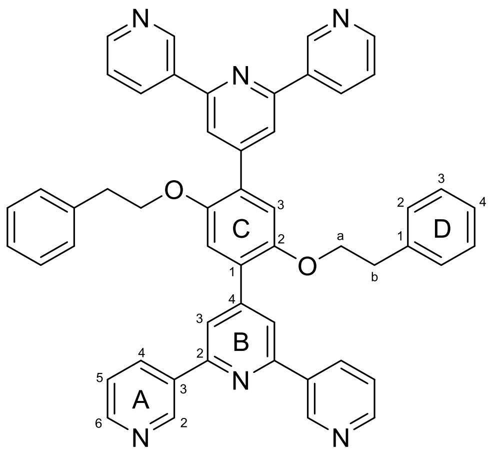

1,4-Bis(3-phenylpropoxy)-2,5-bis(3,2':6',3''-terpyridin-4'-yl)benzene (2)

2,5-Bis(3-phenylpropoxy)terephthalaldehyde (300 mg, 0.745 mmol) was dissolved in EtOH (15 mL), 3-acetylpyridine (451 mg, 0.410 mL, 3.73 mmol) was added to the solution and crushed KOH (209 mg, 3.73 mmol) was added in one portion. Aqueous NH_3 solution (32%, 4.66 mL) was added slowly and the reaction mixture was stirred at room temperature for 5 days. The brownish solid that formed was collected by filtration, washed with water (2 x 20 mL), EtOH (2 x 10 mL) and Et_2O (2 x 20 mL), and then dried *in vacuo* (ca. 24 h). The product was isolated as an off-white powder **2**, 215 mg, 0.266 mmol, 35.7%). M.p. = 203.4-204.6 °C. ^1H NMR (500 MHz, DMSO-d_6): δ/ppm 9.49 (dd, J = 2.3, 0.9 Hz, 4H, H^{A2}), 8.70 (dd, J = 4.7, 1.7 Hz, 4H, H^{A6}), 8.67 (ddd, J = 8.0, 2.3, 1.7 Hz, 4H, H^{A4}), 8.34 (s, 4H, H^{B3}), 7.58 (ddd, J = 8.0, 4.8, 0.9 Hz, 4H, H^{A5}), 7.52 (s, 2H, H^{C3}), 7.13 – 7.05 (m, 6H, H^{D3+D4}), 6.91 (m, 4H, H^{D2}), 4.20 (t, J = 5.9 Hz, 4H, H^{a}), 2.61 (m, 4H, H^{c}), 1.96 (m, 4H, H^{b}). $^{13}C\{1H\}$ NMR (126 MHz, DMSO-d_6) δ/ppm 154.0 (C^{B2}), 150.1 (C^{A6+C2}), 148.1 (C^{A2}), 147.7 (C^{C1}), 141.2 (C^{D1}), 134.4 (C^{A4}), 134.1 (C^{A3}), 128.6 (C^{B4}), 128.2 (C^{D3}), 128.0 (C^{D2}), 125.7 (C^{D4}), 123.9 (C^{A5}), 120.6 (C^{B3}), 115.7 (C^{C3}), 68.3 (C^{a}), 31.8 (C^{c}), 30.8 (C^{b}). UV-Vis ($CHCl_3$, 2.1 × 10^{-5} mol dm^{-3}) λ/nm 280 sh (ε/dm^3 mol^{-1} cm^{-1} 40,850), 316 (19,120). MALDI-TOF-MS (matrix: CHCA) m/z 809.31 $[M+H]^+$ (calc. 809.36). HR-ESI MS m/z 809.3596 $[M+H]^+$ (calc. 809.3599), 405.1841 $[M+2H]^{2+}$ (calc. 405.1836), 270.4589 $[M+3H]^{3+}$ (calc. 270.4581). Found C 79.52, H 5.49, N 9.98; required for $C_{54}H_{44}N_6O_2$: C 80.17, H 5.48, N 10.39.

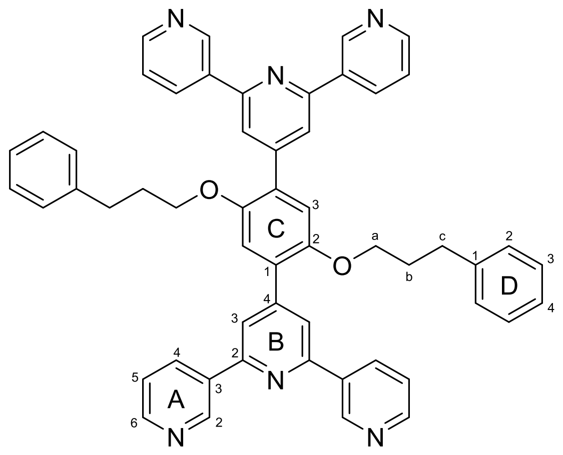

$[Cu_2(hfacac)_4(\mathbf{1})]_n \cdot 3.6n(1,2\text{-}Cl_2C_6H_4) \cdot 2nCHCl_3$

A 1,2-dichlorobenzene (8 mL) solution of $[Cu(hfacac)_2] \cdot H_2O$ (12 mg, 0.024 mmol) was layered over a $CHCl_3$ solution (5 mL) of **1** (7.8 mg, 0.010 mmol) in a crystallization tube (i.d. = 13.6 mm, vol. = 24 mL) sealed with a septum. Light green plate-like crystals visible to the eye were first obtained after 17 days, and a single crystal was selected for X-ray diffraction. The remaining crystals were analysed by powder X-ray diffraction (PXRD) and FT-IR spectroscopy.

$[Zn_2(hfacac)_4(\mathbf{1})]_n \cdot nMeC_6H_5 \cdot 1.8nCHCl_3$

A toluene (8 mL) solution of $[Zn(hfacac)_2] \cdot 2H_2O$ (13 mg, 0.025 mmol) was layered over a $CHCl_3$ solution (5 mL) of **1** (7.8 mg, 0.010 mmol) in a crystallization tube (i.d. = 13.6 mm, vol. = 24 mL) sealed with a septum. Colourless block-like crystals visible to the eye were first obtained after 1 month, and a single crystal was selected for X-ray diffraction. The remaining crystals were analysed by powder X-ray diffraction (PXRD).

$[Cu_2(hfacac)_4(\mathbf{2})]_n \cdot nMeC_6H_5 \cdot 2nH_2O$

A toluene (8 mL) solution of $[Cu(hfacac)_2] \cdot H_2O$ (12 mg, 0.024 mmol) was layered over a $CHCl_3$ solution (5 mL) of **2** (8.1 mg, 0.010 mmol) in a crystallization tube (i.d. = 13.6 mm, vol. = 24 mL) sealed with a septum. Light green block-like crystals visible to the eye were first obtained after 1 month, and a single crystal was selected for X-ray diffraction. The remaining crystals were analysed by powder X-ray diffraction (PXRD) and FT-IR spectroscopy.

$[Cu_2(hfacac)_4(\mathbf{2})]_n \cdot 2.8nC_6H_5Cl$

A chlorobenzene (8 mL) solution of $[Cu(hfacac)_2] \cdot H_2O$ (12 mg, 0.024 mmol) was layered over a $CHCl_3$ solution (5 mL) of **2** (8.1 mg, 0.010 mmol) in a crystallization tube (i.d. = 13.6 mm, vol. = 24 mL) sealed with a septum. Light green block-like crystals visible to the eye were first obtained after 1 month, and

a single crystal was selected for X-ray diffraction. The remaining crystals were analysed by powder X-ray diffraction (PXRD) and FT-IR spectroscopy.

$[Cu_2(hfacac)_4(2)]_n$·2*n*(1,2-$Cl_2C_6H_4$)·0.4*n*$CHCl_3$·0.5*n*H_2O

A 1,2-dichlorobenzene (8 mL) solution of $[Cu(hfacac)_2]$·H_2O (12 mg, 0.024 mmol) was layered over a $CHCl_3$ solution (5 mL) of **2** (8.1 mg, 0.010 mmol) in a crystallization tube (i.d. = 13.6 mm, vol. = 24 mL) sealed with a septum. Light green plate-like crystals visible to the eye were first obtained after 1 month, and a single crystal was selected for X-ray diffraction. The remaining crystals were analysed by powder X-ray diffraction (PXRD) and FT-IR spectroscopy.

Crystallography

Single crystal data for **1a** and $[Cu_2(hfacac)_4(\mathbf{1})]_n$·3.6*n*(1,2-$Cl_2C_6H_4$)·2*n*$CHCl_3$ were collected on a STOE StadiVari diffractometer equipped with a Metaljet D2 source (GaKα radiation) and a Pilatus300K detector. Crystallographic data for **2a** were collected using a Bruker APEX-II CCD diffractometer with CuKα radiation. Single crystal data of $[Zn_2(hfacac)_4(\mathbf{1})]_n$·*n*$MeC_6H_5$·1.8*n*$CHCl_3$ was collected with CuKα radiation on an Agilent SuperNova, Dual-source, with an Atlas detector. For $[Cu_2(hfacac)_4(\mathbf{2})]_n$·*n*$MeC_6H_5$·2*n*$H_2O$, $[Cu_2(hfacac)_4(\mathbf{2})]_n$·2.8*n*$C_6H_5Cl$ and $[Cu_2(hfacac)_4(\mathbf{2})]_n$·2*n*(1,2-$Cl_2C_6H_4$)·0.4*n*$CHCl_3$·0.5*n*$H_2O$, data collection was performed on a Rigaku-Oxford Diffraction XtaLAB Synergy-S dual source diffractometer equipped with a Cu and Mo PhotonJet microfocus X-ray sources and a Dectris Pilatus3 R 200K HPC detector. The structures were solved using ShelXT v. 2018/2[2] and Olex2[3], and the model was refined with ShelXL v. 2018/3.[4] All H atoms were included at geometrically calculated positions. C(H) and C(H,H) groups were refined using a riding model with U_{iso} = 1.2 of the parent atom, while in the case of the group C(H,H,H) U_{iso} is 1.5. Structure analysis used CSD Mercury 2021.3.0.[5] In $[Cu_2(hfacac)_4(\mathbf{1})]_n$·3.6*n*(1,2-$Cl_2C_6H_4$)·2*n*$CHCl_3$, a CF_3 group of the polymer and 1,2-dichlorobenzene solvent molecules were disordered and were refined with geometrical constraints and constraints on their thermal parameters. One CF_3 of one the $[hfacac]^-$ ligand in $[Zn_2(hfacac)_4(\mathbf{1})]_n$·*n*$MeC_6H_5$·1.8*n*$CHCl_3$ was disordered over 2 orientations. The central phenylene spacer with its 3-phenylethoxy substituent in $[Zn_2(hfacac)_4(\mathbf{1})]_n$·*n*$MeC_6H_5$·1.8*n*$CHCl_3$ was disordered in a 1:1 ratio. In the absence of the phenyl moiety, once a toluene and once a chloroform molecule were present instead. The toluene molecule was further rotationally disordered; the position of the disordered methyl group could only be identified for half of the half occupied toluene molecule. Geometrical constraints and restraints on their thermal parameters were used to model them. In $[Cu_2(hfacac)_4(\mathbf{2})]_n$·*n*$MeC_6H_5$·2*n*$H_2O$ and $[Cu_2(hfacac)_4(\mathbf{2})]_n$·2.8*n*$C_6H_5Cl$, both solvent and one $[hfacac]^-$ ligand were disordered requiring geometrical restraints and restraints on their thermal parameters in the modelling process. All the $[hfacac]^-$ ligands in $[Cu_2(hfacac)_4(\mathbf{2})]_n$·2*n*(1,2-$Cl_2C_6H_4$)·0.4*n*$CHCl_3$·0.5*n*$H_2O$ were disordered over two positions with 1:1 ratio. Geometrical constraints for the aromatic ring and restraints for the thermal parameters had to be used to treat the solvent molecules. In the structural discussions, only the major (or one of the equal) occupancy sites are considered in each disordered entity. Some structures show relative high R_1/R_2 values, which is not unexpected, given the disorder present in some metal complexes and co-solvate molecules.

PXRD data were collected at room temperature in transmission mode using a Stoe Stadi P diffractometer, equipped with CuKα1 radiation (Ge(111) monochromator) and a DECTRIS MYTHEN 1K detector. Whole-pattern decomposition (profile matching) analysis of the diffraction patterns was done using either overlay in Excel or the package FULLPROF SUITE (v. September 2020)[6, 7] using a previously determined instrument resolution function based on a NIST640d standard. The structural

models were derived from the single crystal X-ray diffraction data. When FULLPROF SUITE was used, refined parameters in Rietveld were scale factor, zero shift, lattice parameters, Cu and halogen atomic positions, background points, and peaks shapes as a Thompson Cox Hastings pseudo-Voigt function. Preferred orientations as a March–Dollase multi-axial phenomenological model were incorporated into the analysis.

References

1. G. Manfroni, A. Prescimone, E. C. Constable and C. E. Housecroft, *Crystals*, 2021, **11**, 325.
2. G. Sheldrick, *Acta Crystallogr.*, 2015, **A71**, 3-8.
3. O. V. Dolomanov, L. J. Bourhis, R. J. Gildea, J. A. K. Howard and H. Puschmann, *J. Appl. Crystallogr.*, 2009, **42**, 339-341.
4. G. Sheldrick, *Acta Crystallogr.*, 2015, **C71**, 3-8.
5. C. F. Macrae, I. Sovago, S. J. Cottrell, P. T. A. Galek, P. McCabe, E. Pidcock, M. Platings, G. P. Shields, J. S. Stevens, M. Towler and P. A. Wood, *J. Appl. Crystallogr.*, 2020, **53**, 226-235.
6. J. Rodríguez-Carvajal, *Physica B Condens. Matter*, 1993, **192**, 55-69.
7. T. Roisnel and J. Rodríguez-Carvajal, in *In Materials Science Forum. Proceedings of the European Powder Diffraction Conf. (EPDIC 7)*, Barcelona, Spain, 2001, pp. 118-123.

Supplementary Figures

Figures S1–S12: NMR spectra of compounds 1a–2a and 1–2.

Figures S13–S16: ATR-IR spectra of compounds 1a–2a and 1–2.

Figures S17: UV-vis spectra of compounds 1a–2a and 1–2.

Figures S18–S21: MALDI-TOF mass spectra of compounds 1a–2a and 1–2.

Figures S22–S23: HR-ESI mass spectra of compounds 1–2.

Figures S24–S36: Structural figures and PXRD.

Figures S37–S40: ATR-IR spectra of the coordination polymers.

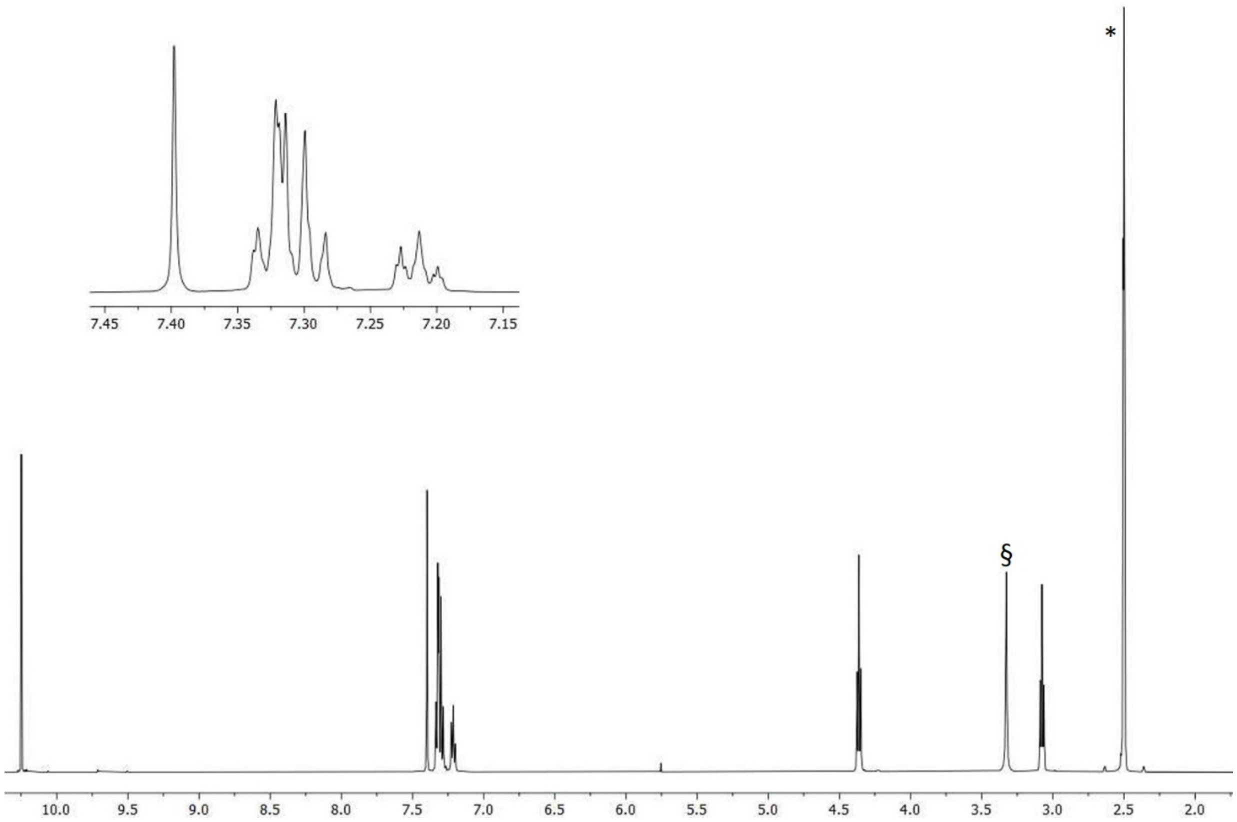

Figure S1: ^{1}H NMR spectrum (500 MHz, DMSO-d_6, 298 K) of **1a**. * = DMSO-d_5, § = H_2O

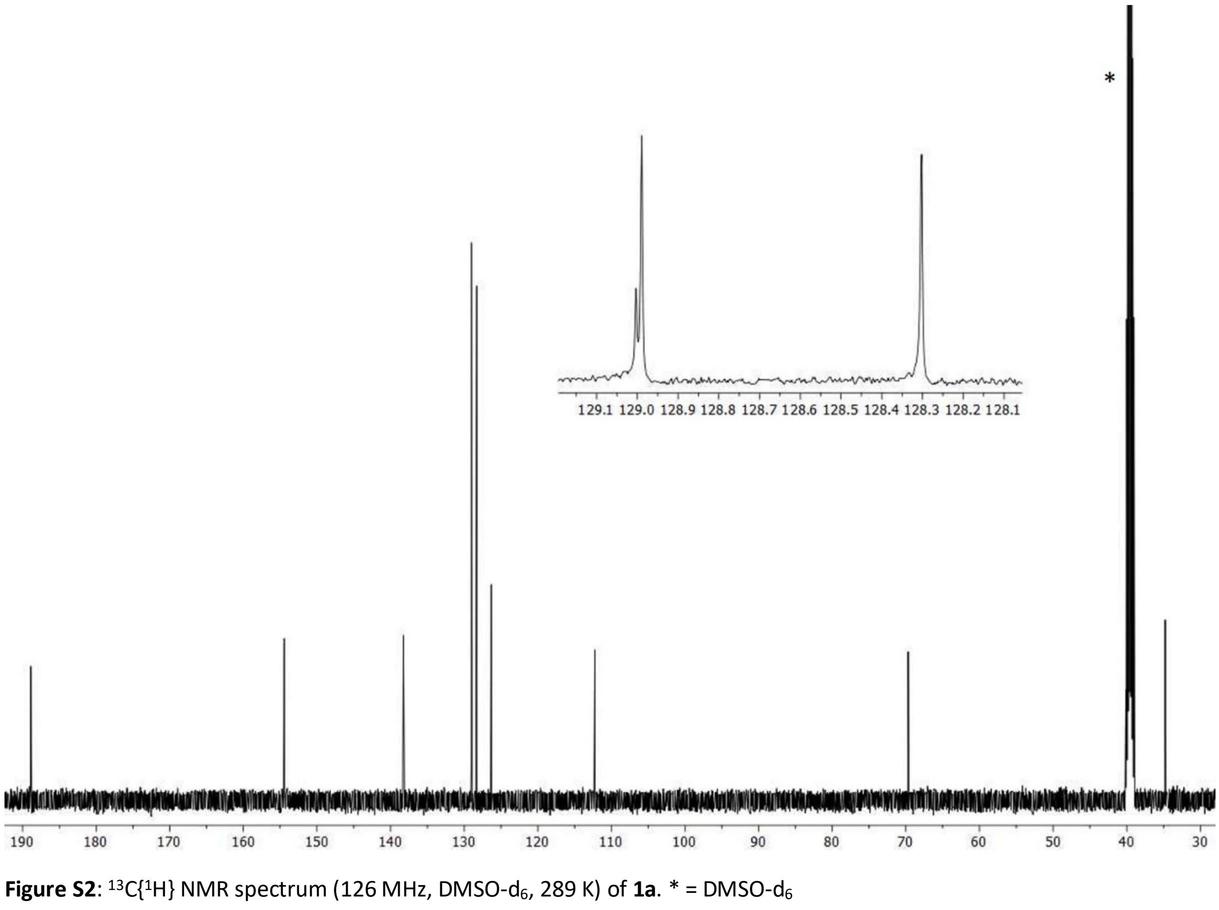

Figure S2: ^{13}C{^{1}H} NMR spectrum (126 MHz, DMSO-d_6, 289 K) of **1a**. * = DMSO-d_6

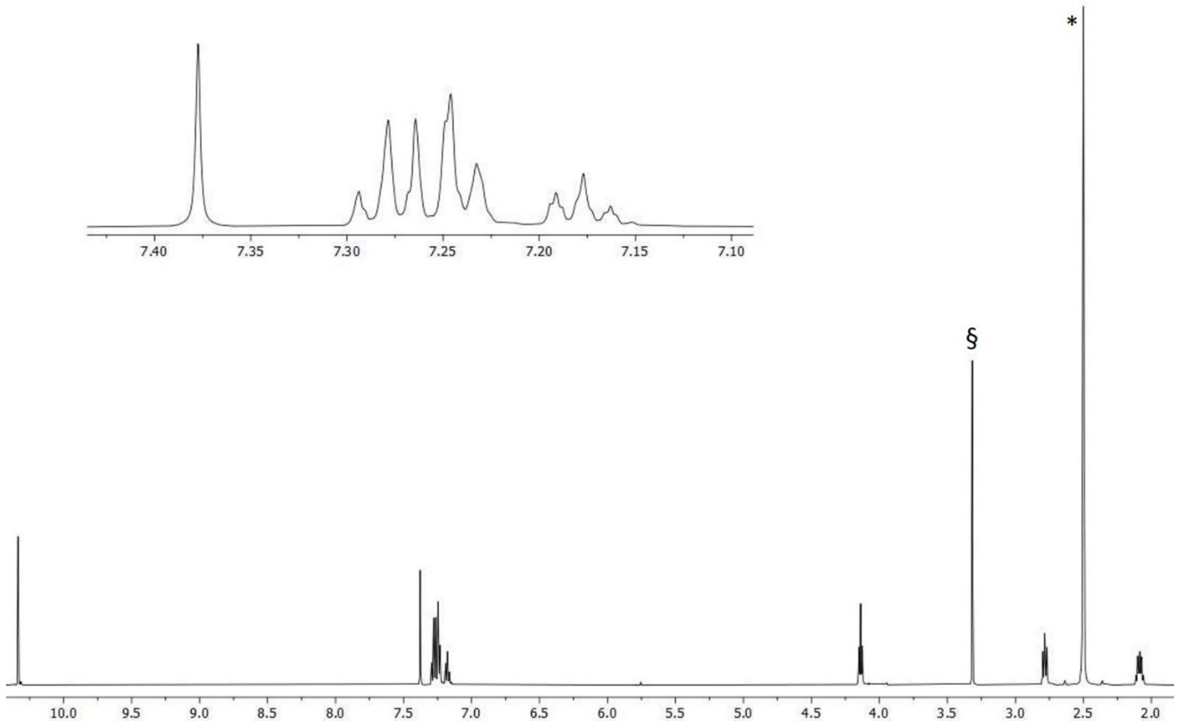

Figure S3: ^{1}H NMR spectrum (500 MHz, DMSO-d_6, 298 K) of **2a**. * = DMSO-d_5, § = H_2O

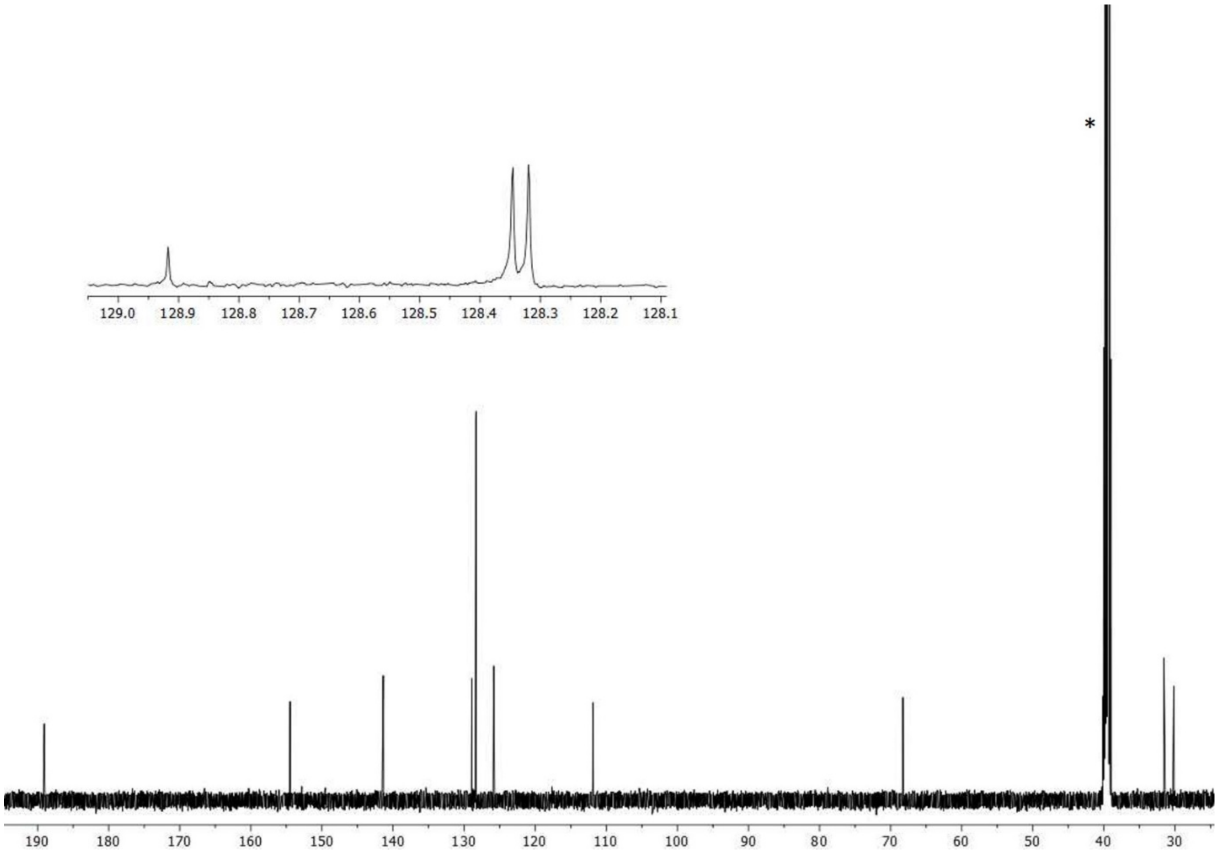

Figure S4: $^{13}C\{^{1}H\}$ NMR spectrum (126 MHz, DMSO-d_6, 289 K) of **2a**. * = DMSO-d_6

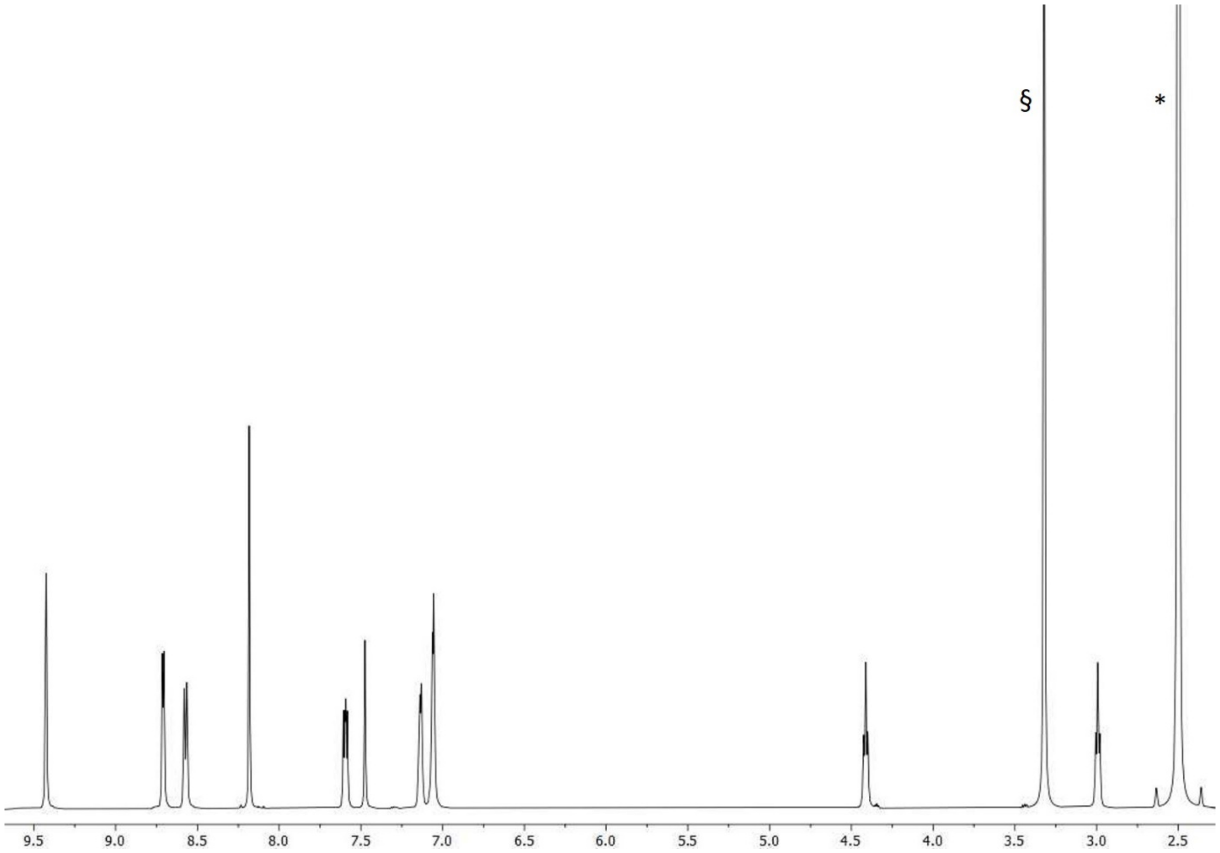

Figure S5: ^1H NMR spectrum (500 MHz, DMSO-d_6, 298 K) of **1**. * = DMSO-d_5, § = H_2O

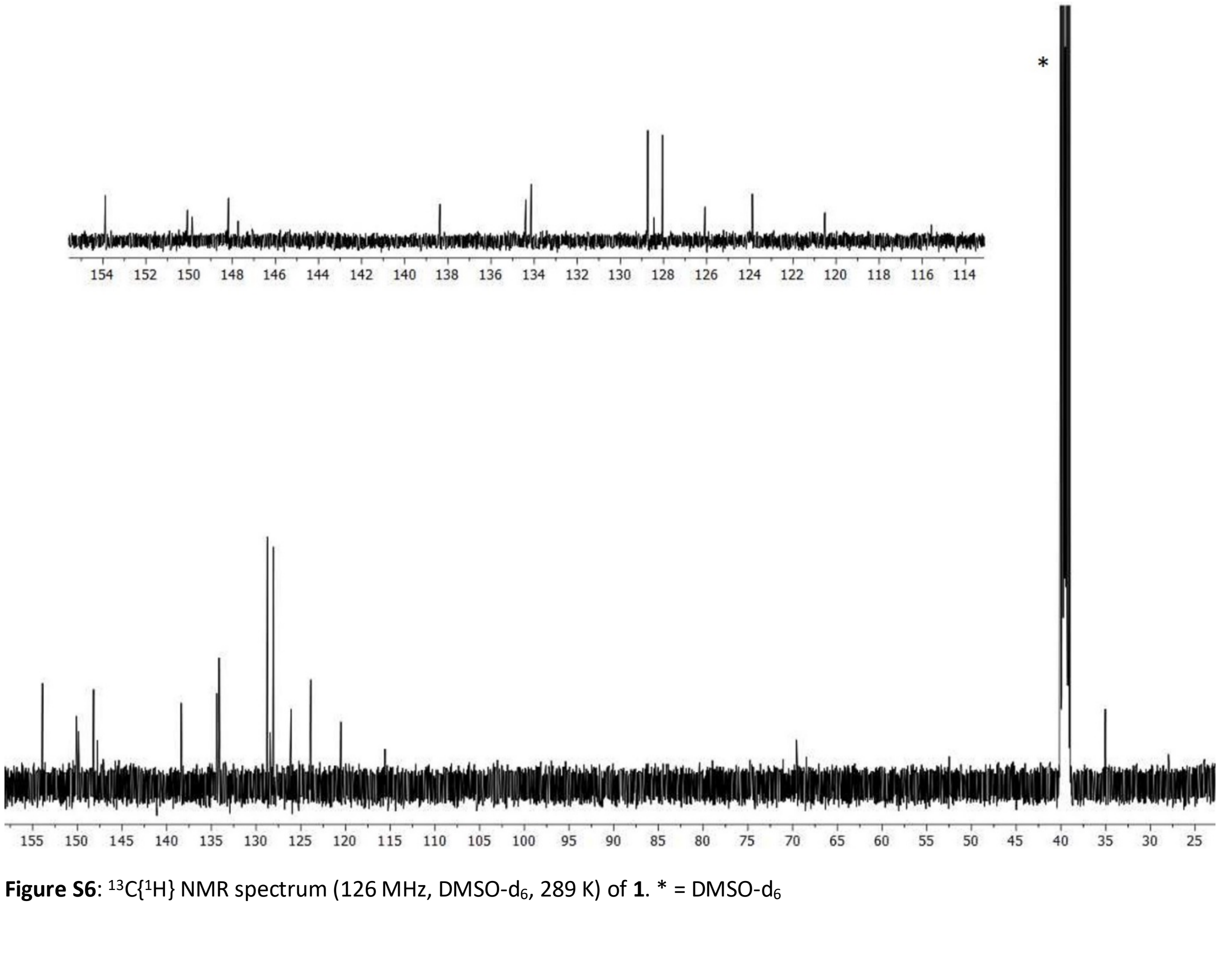

Figure S6: $^{13}C\{^1H\}$ NMR spectrum (126 MHz, DMSO-d_6, 289 K) of **1**. * = DMSO-d_6

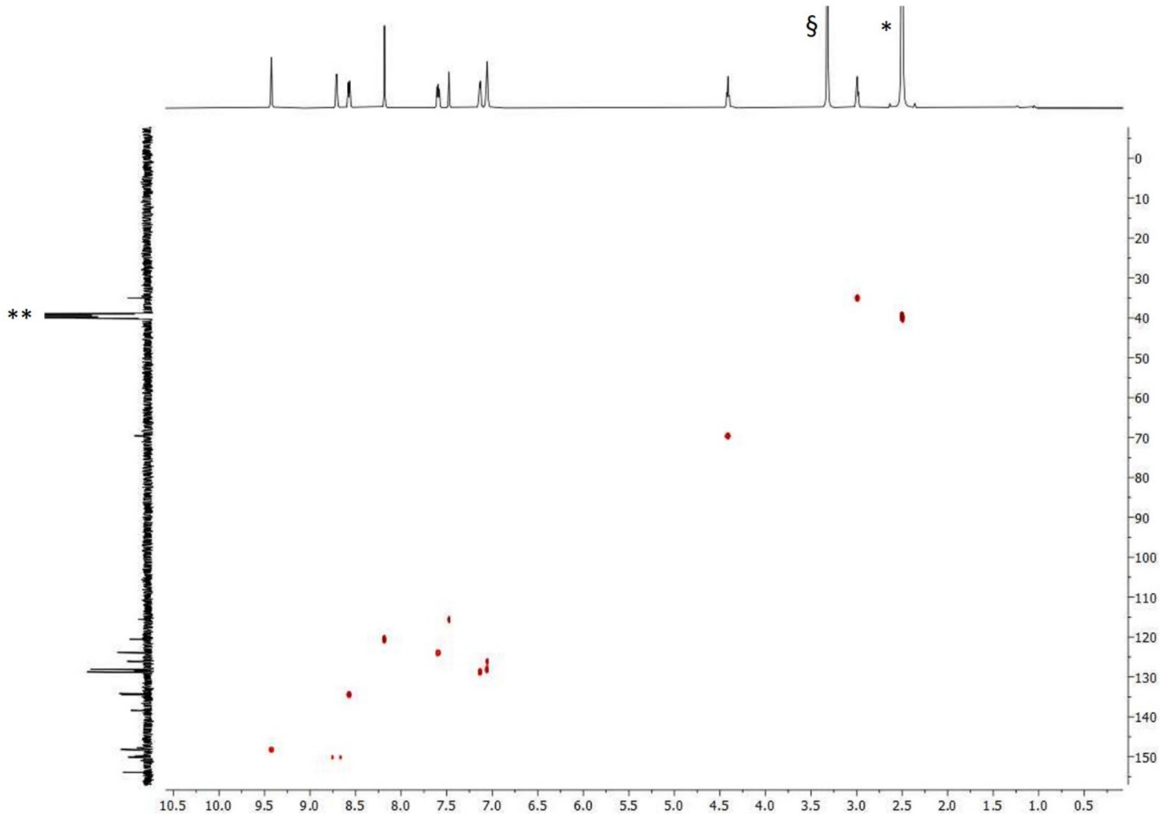

Figure S7: HMQC spectrum (^1H 500 MHz, $^{13}C\{^1H\}$ 126 MHz, DMSO-d_6, 298 K) of **1**. * = DMSO-d_5, ** = DMSO-d_6, § = H_2O

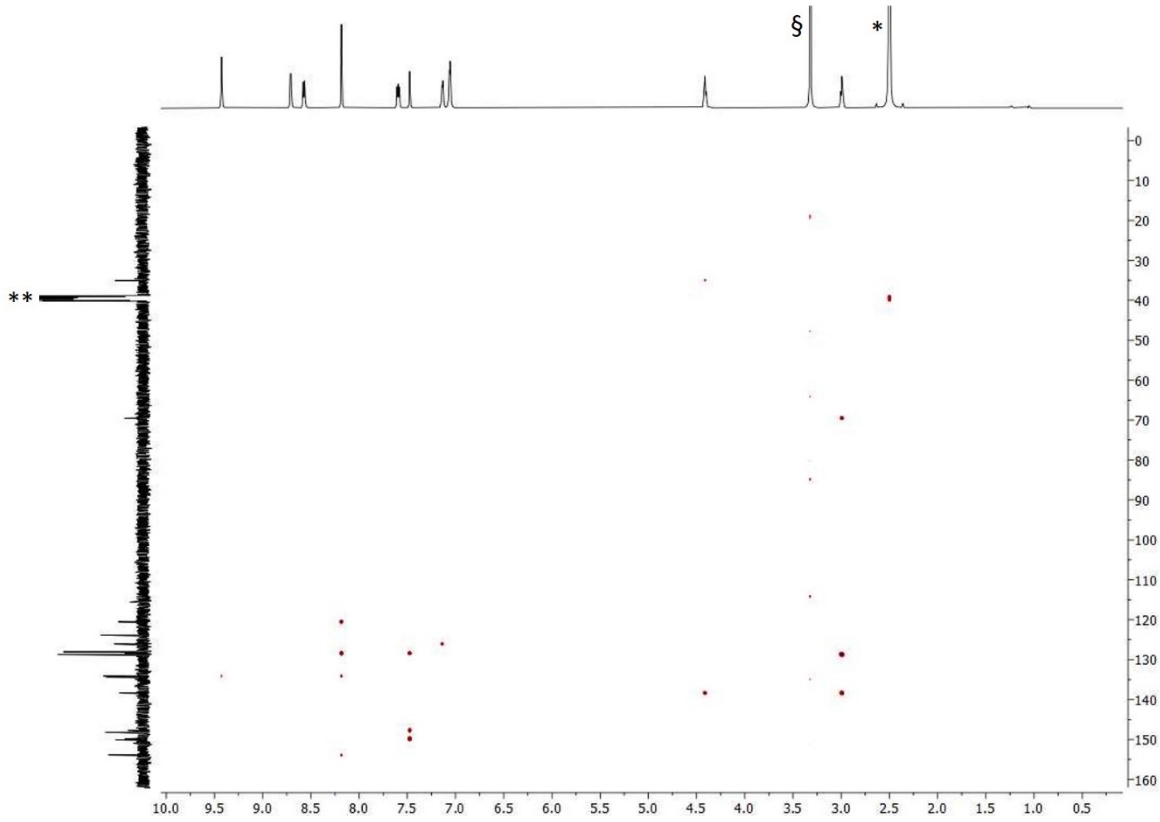

Figure S8: HMBC spectrum (^1H 500 MHz, $^{13}C\{^1H\}$ 126 MHz, DMSO-d_6, 298 K) of **1**. * = DMSO-d_5, ** = DMSO-d_6, § = H_2O

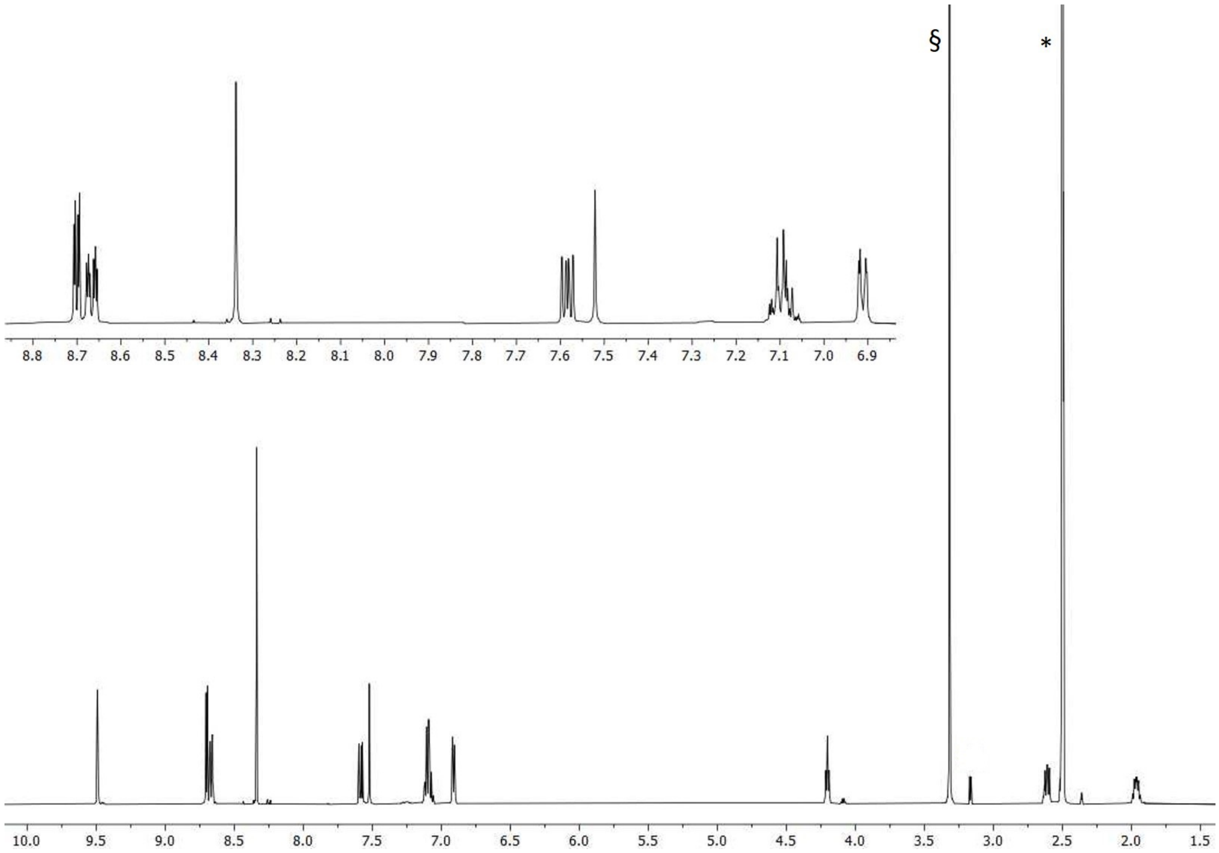

Figure S9: ^{1}H NMR spectrum (500 MHz, DMSO-d_6, 298 K) of **2**. * = DMSO-d_5, § = H_2O

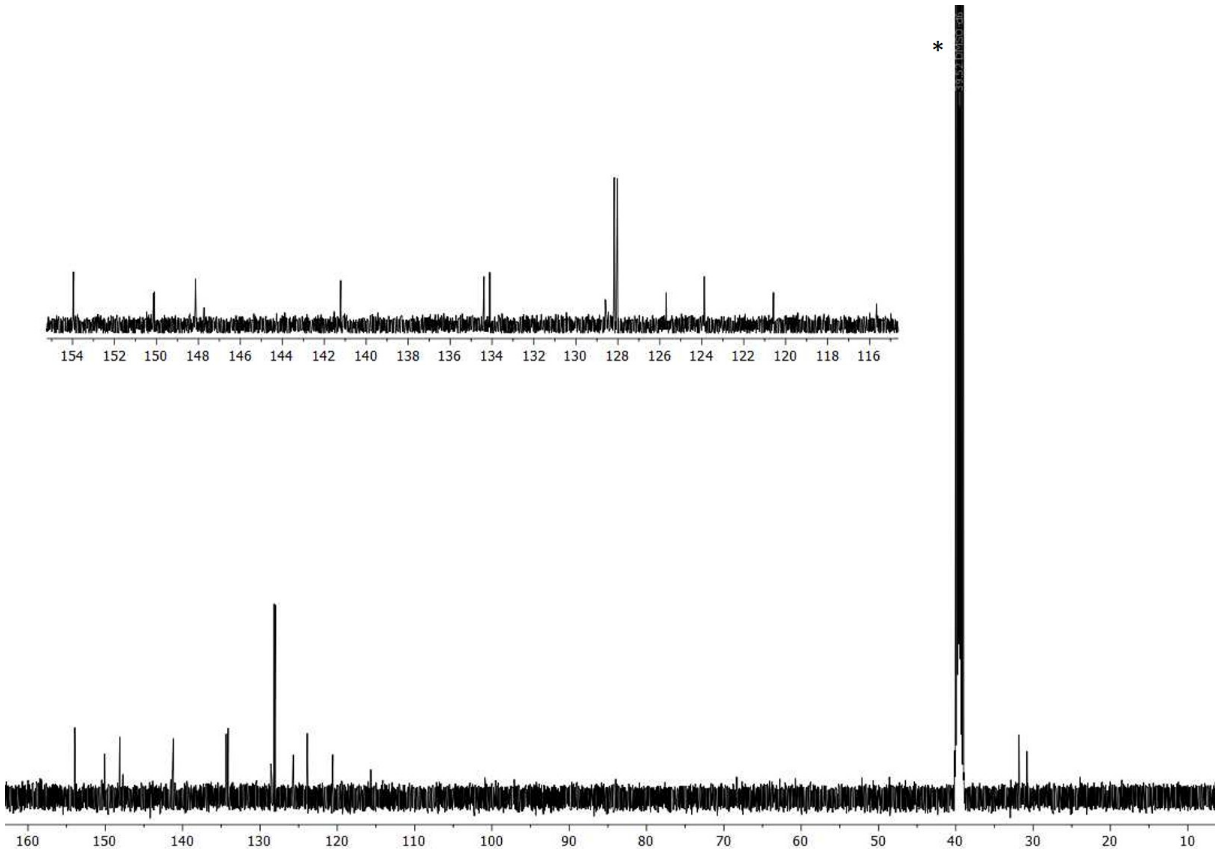

Figure S10: $^{13}C\{^{1}H\}$ NMR spectrum (126 MHz, DMSO-d_6, 289 K) of **2**. * = DMSO-d_6

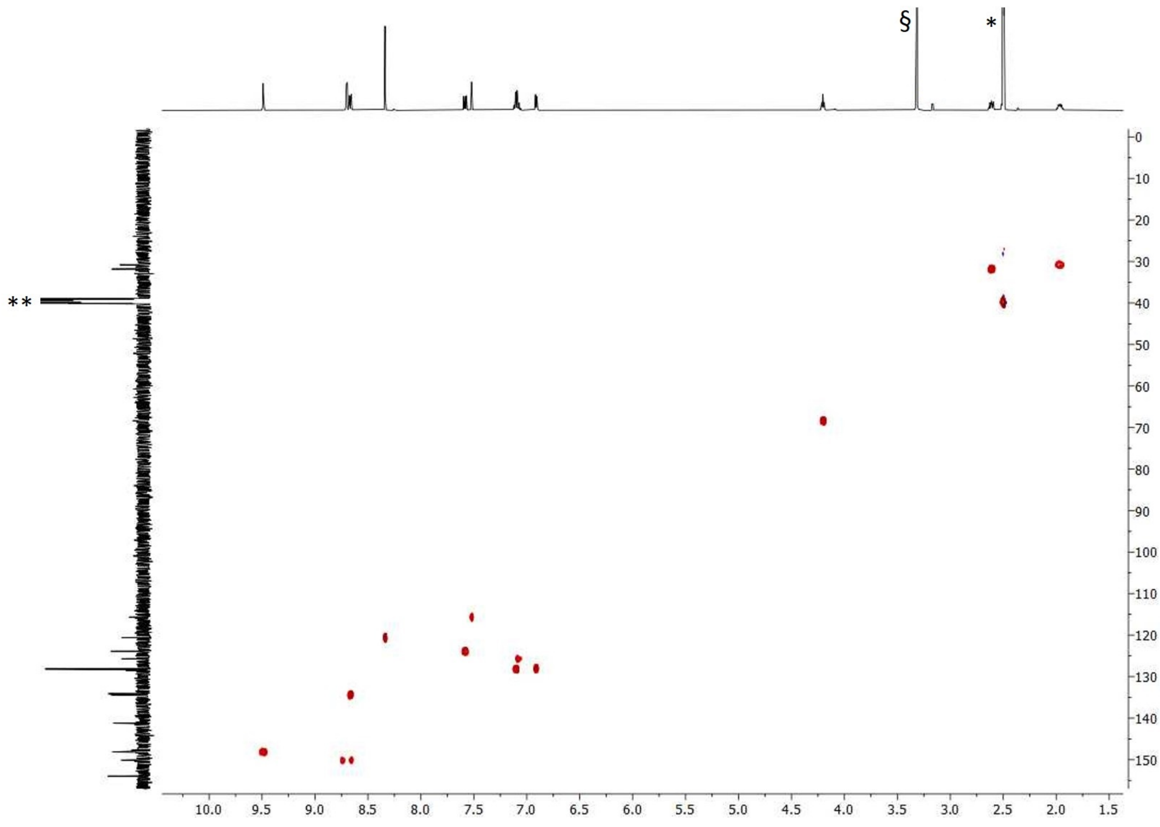

Figure S11: HMQC spectrum (^1H 500 MHz, $^{13}C\{^1H\}$ 126 MHz, DMSO-d_6, 298 K) of **2**. * = DMSO-d_5, ** = DMSO-d_6, § = H_2O

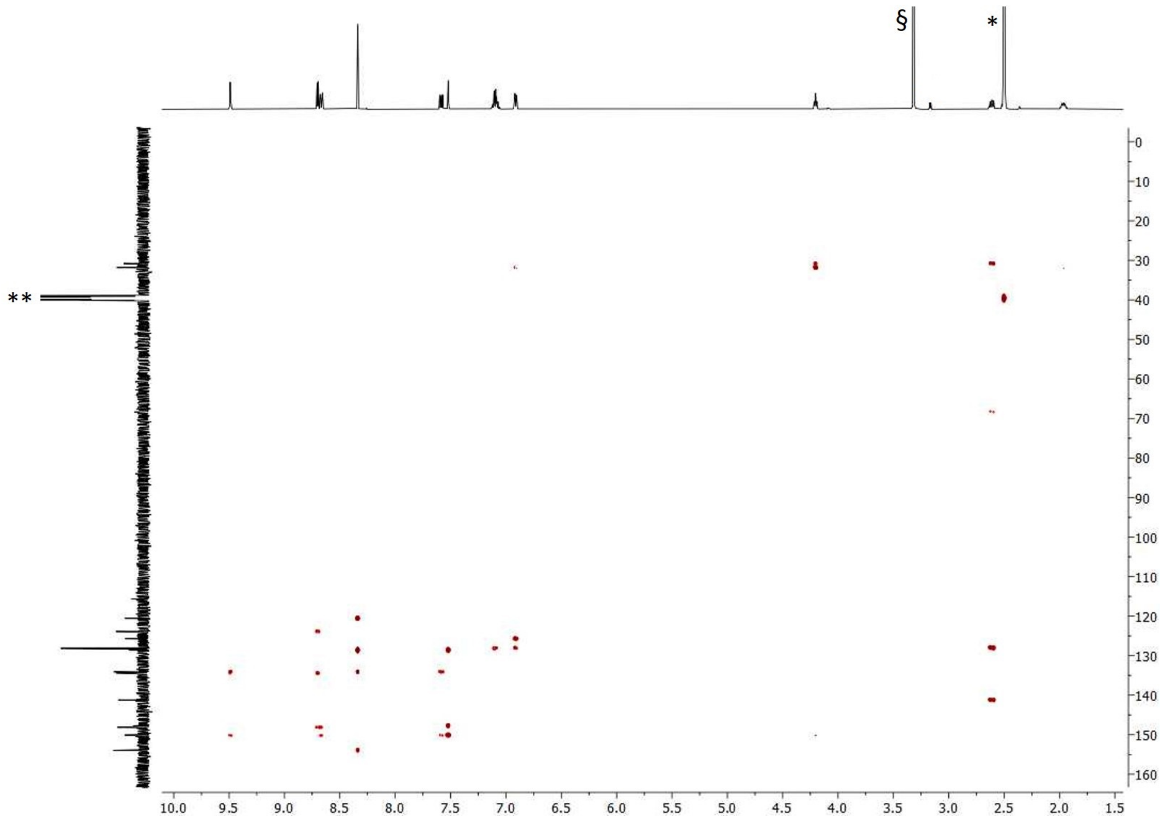

Figure S12: HMBC spectrum (^1H 500 MHz, $^{13}C\{^1H\}$ 126 MHz, DMSO-d_6, 298 K) of **2**. * = DMSO-d_5, ** = DMSO-d_6, § = H_2O

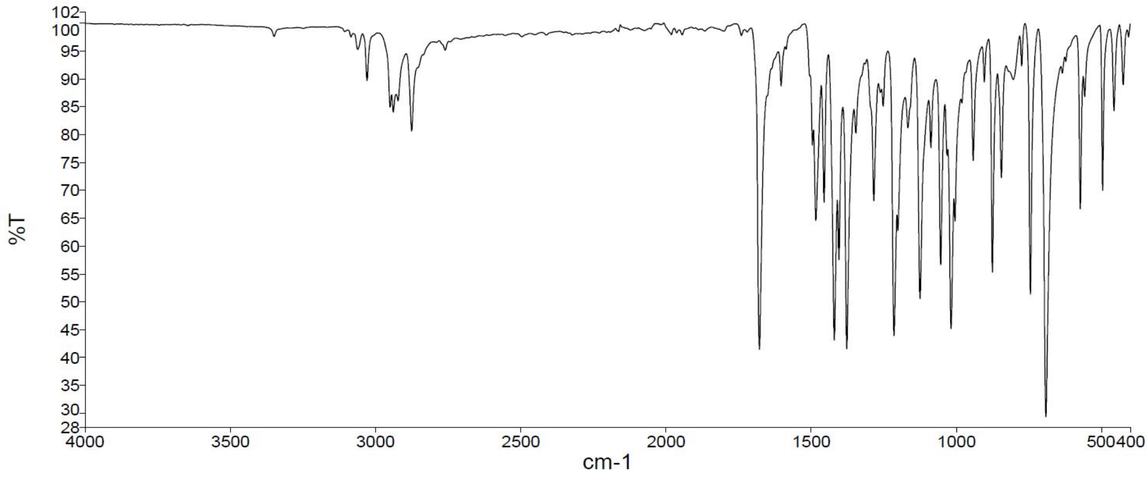

Figure S13: The solid state FT-IR spectrum of **1a**.

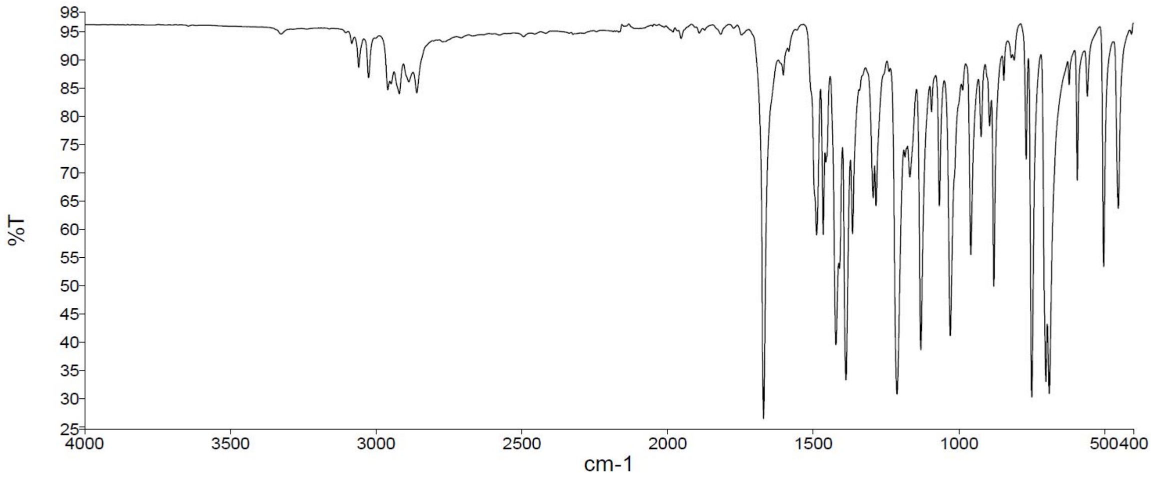

Figure S14: The solid state FT-IR spectrum of **2a**.

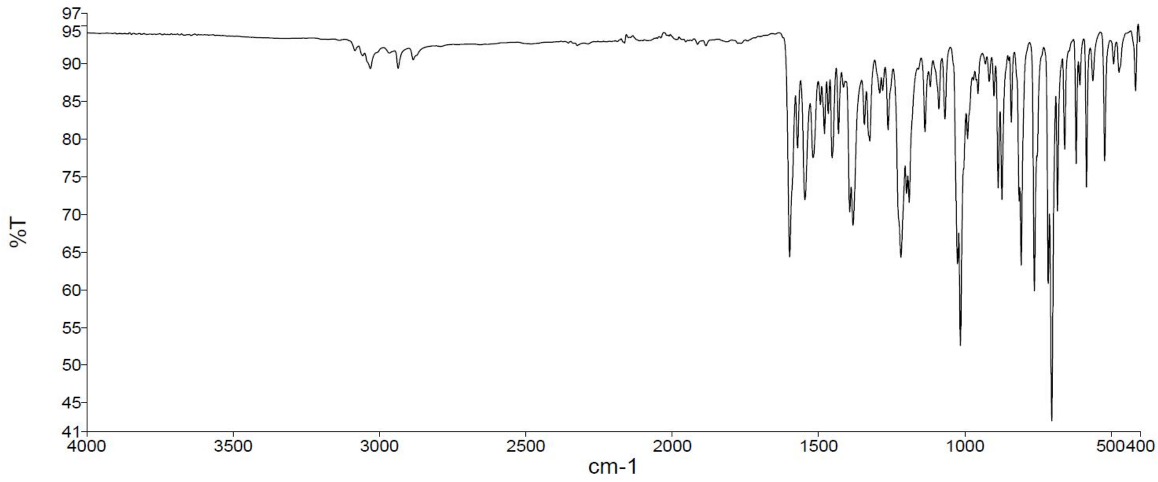

Figure S15: The solid state FT-IR spectrum of **1**.

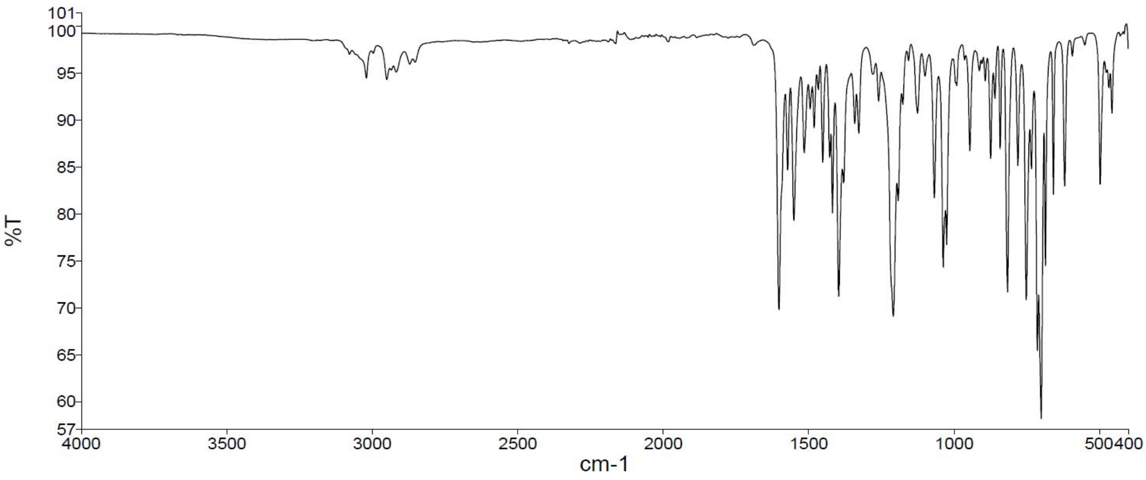

Figure S16: The solid state FT-IR spectrum of **2**.

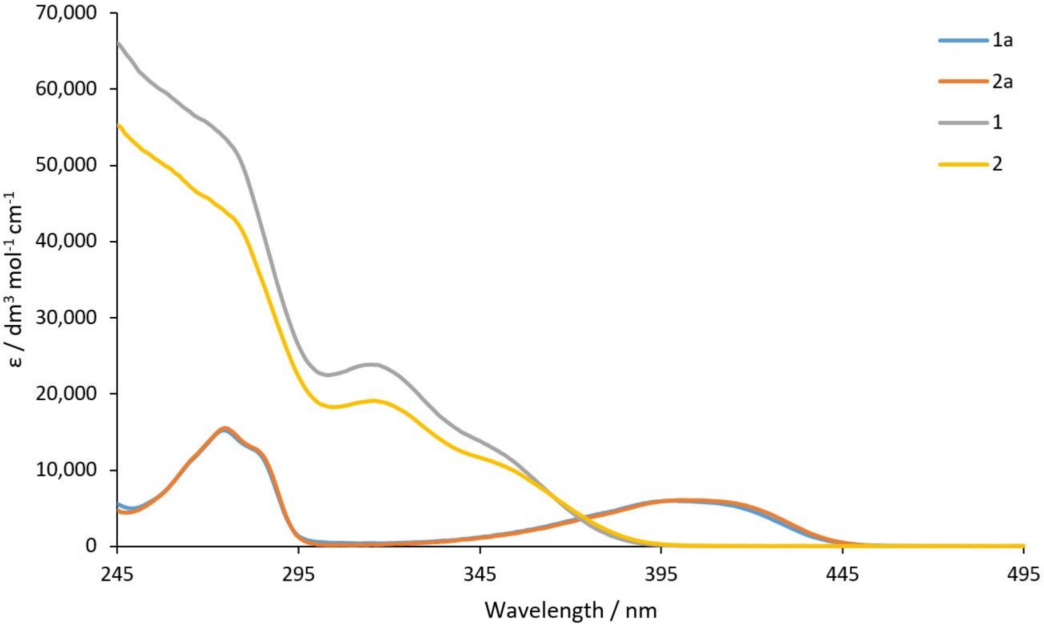

Figure S17: Solution absorption spectra of **1a**–**2a** and **1-2** in $CHCl_3$ (2×10^{-5} mol dm^{-3}).

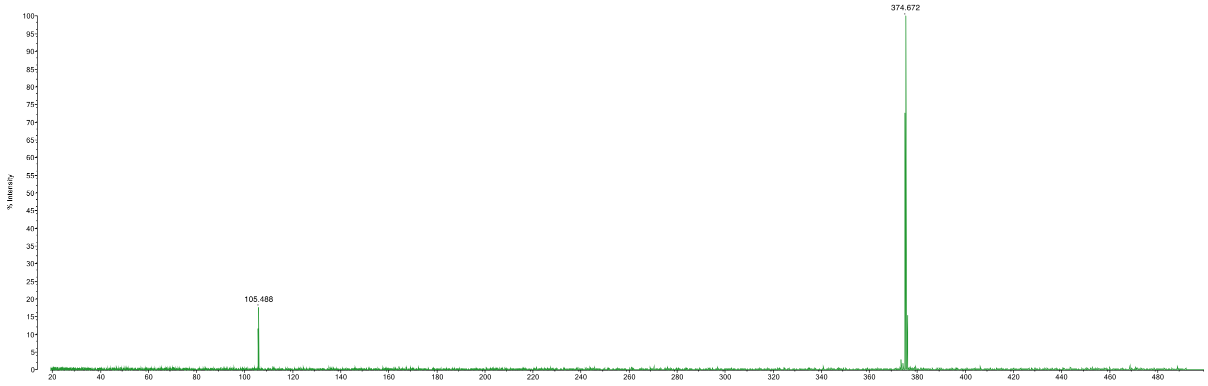

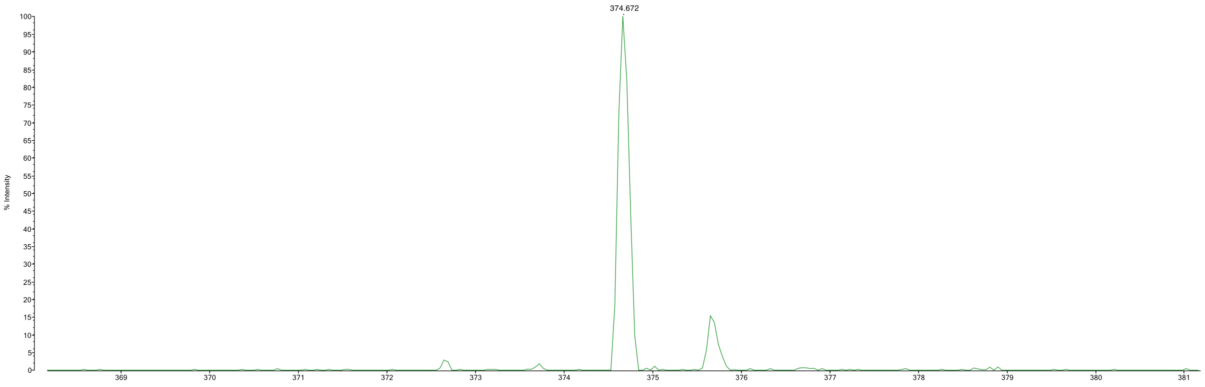

Figure S18: The MALDI-TOF mass spectrum of **1a**, and expansion of the base peak.

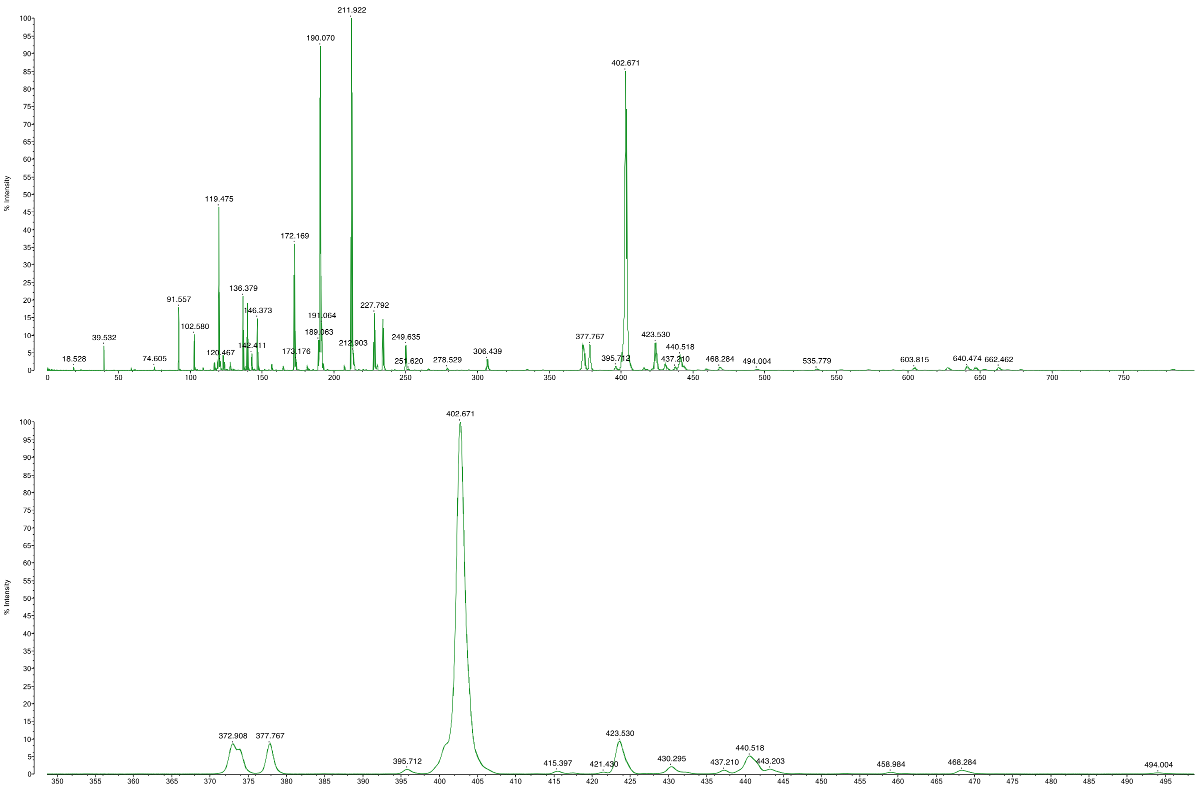

Figure S19: The MALDI-TOF mass spectrum of **2a**, and expansion of the $[M]^+$ peak.

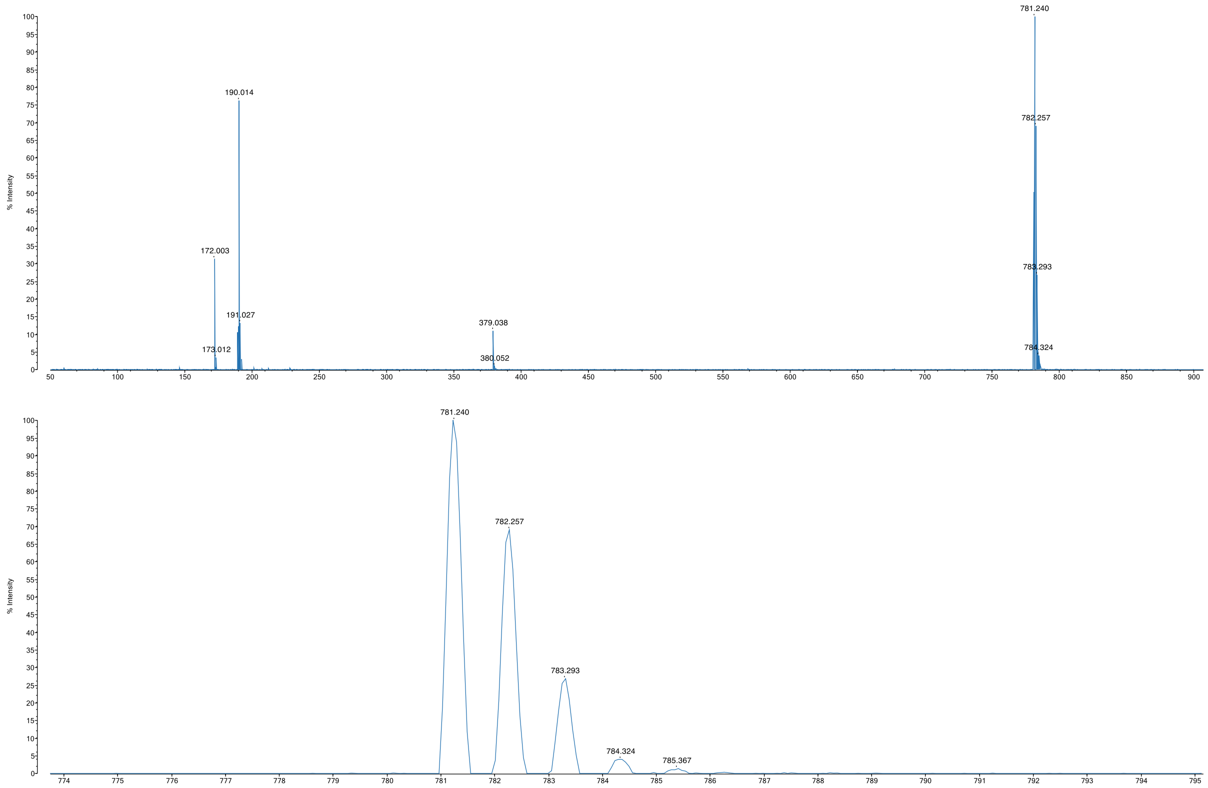

Figure S20: The MALDI-TOF mass spectrum of **1**, and expansion of the base peak.

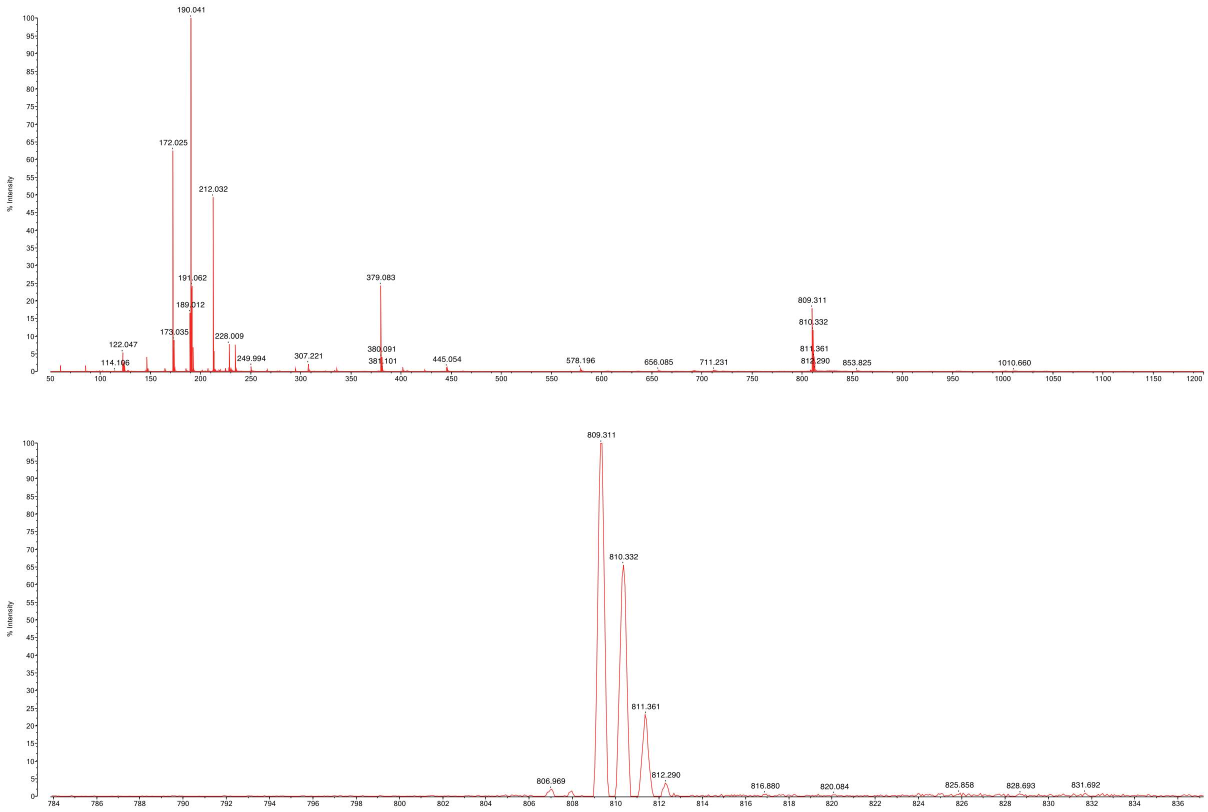

Figure S21: The MALDI-TOF mass spectrum of **2**, and expansion of the $[M+H]^+$ peak.

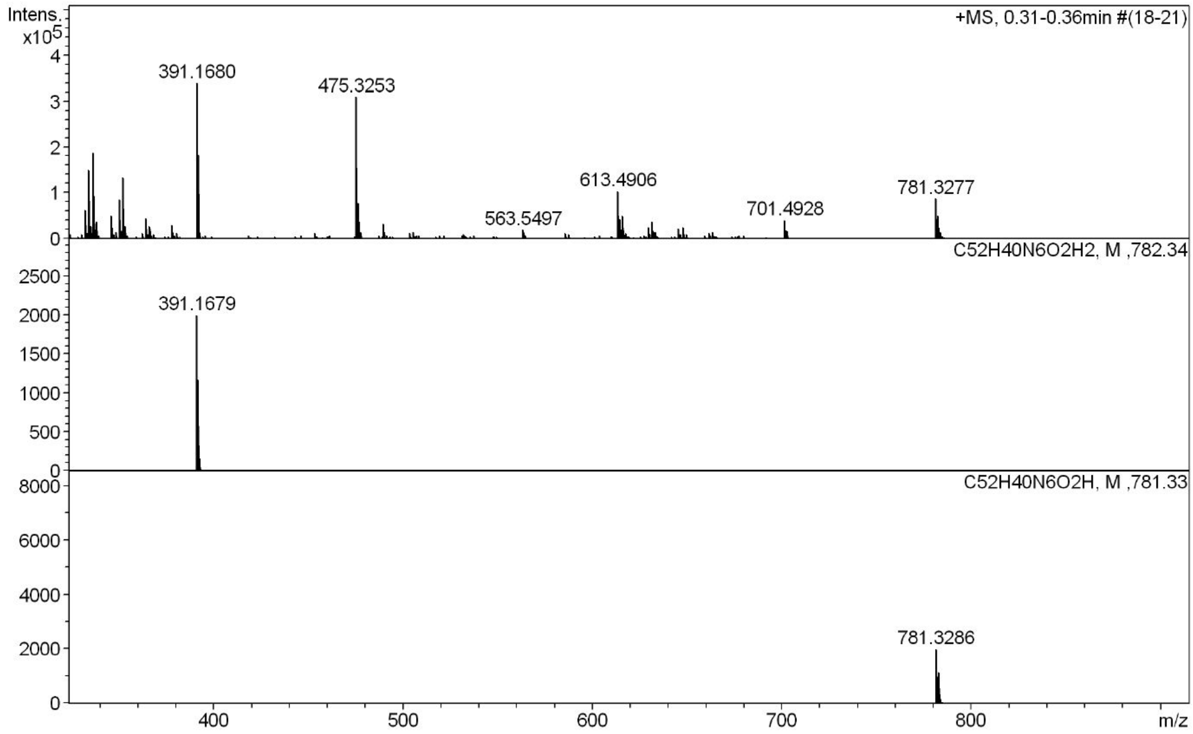

Figure S22: HR-ESI mass spectrum of **1** comparing the experimental isotope pattern for the base peak arising from $[M+2H]^{2+}$ and the $[M+H]^{+}$ peak (top) with the calculated isotope pattern (middle, $[M+2H]^{2+}$; bottom, $[M+H]^{+}$).

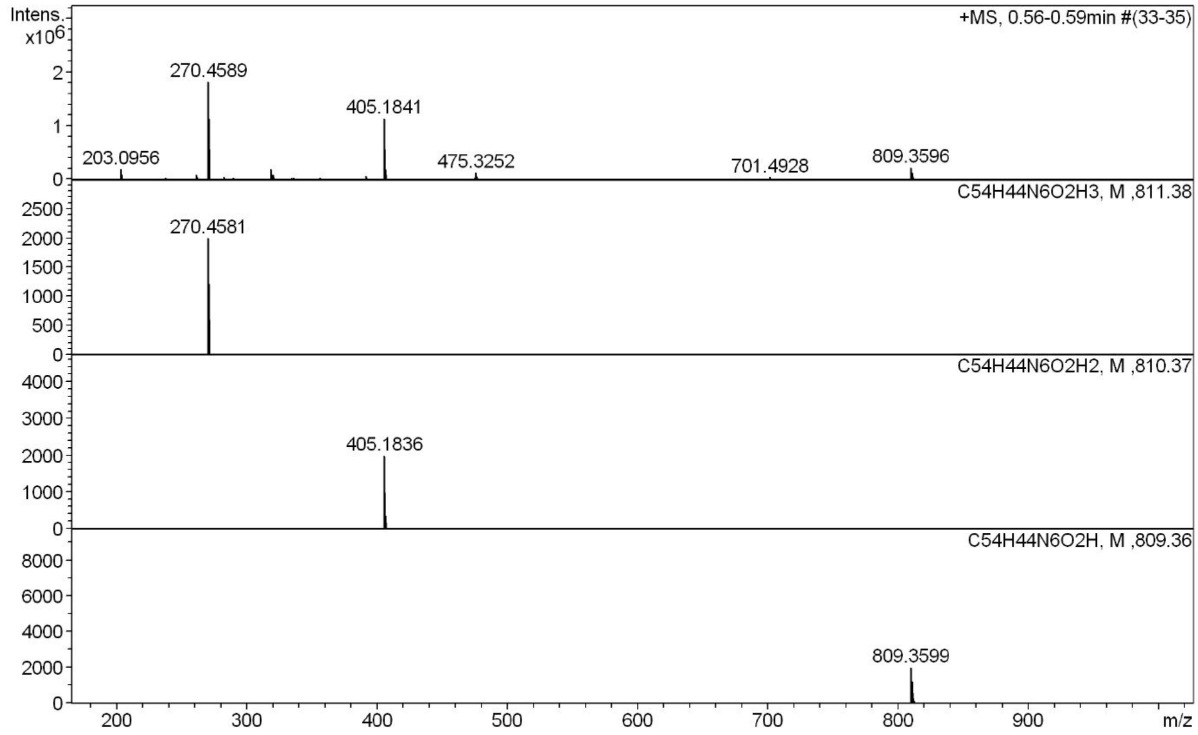

Figure S23: HR-ESI mass spectrum of **2** comparing the experimental isotope pattern for the peaks arising from $[M+3H]^{3+}$, $[M+2H]^{2+}$ and $[M+H]^{+}$ (top) with the calculated isotope pattern (second plot, $[M+3H]^{3+}$; third plot, $[M+2H]^{2+}$; fourth plot, $[M+H]^{+}$).

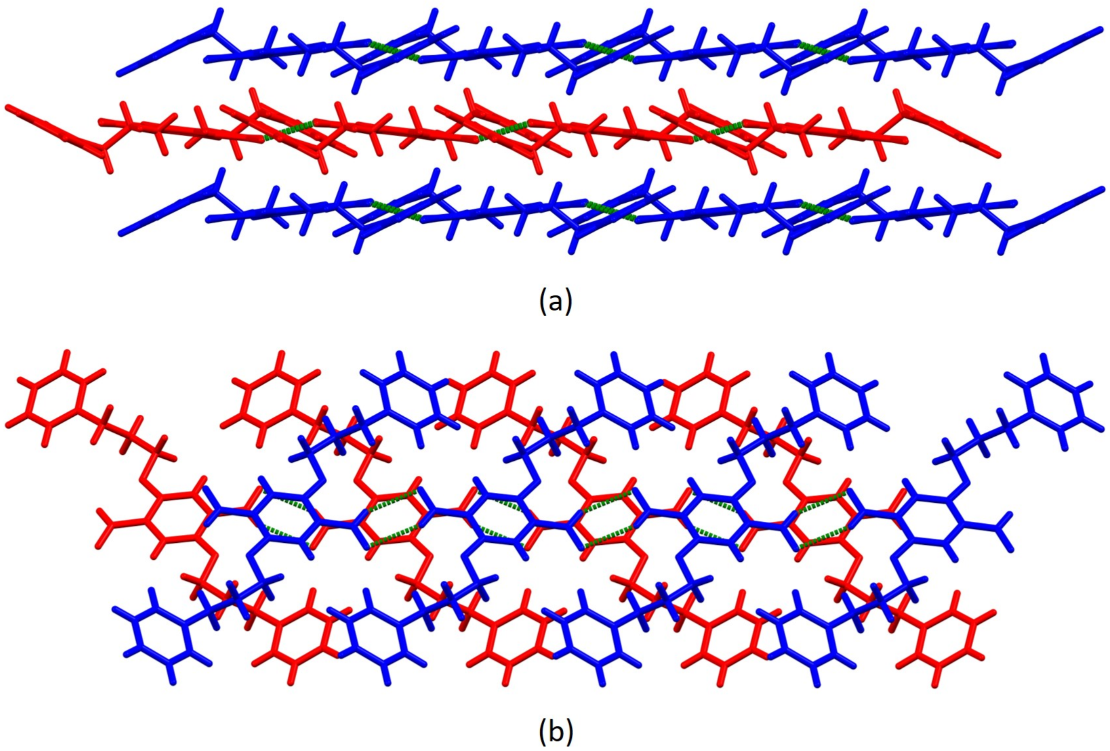

Figure S24: (a) Looking along the side of the ribbons in **2a** to illustrate that the packing is following an ABAB pattern. (b) Ribbons are shown down on the stack. The intermolecular O...H–C hydrogen bonds are illustrated in green and the individual ribbons are alternately coloured in red and blue.

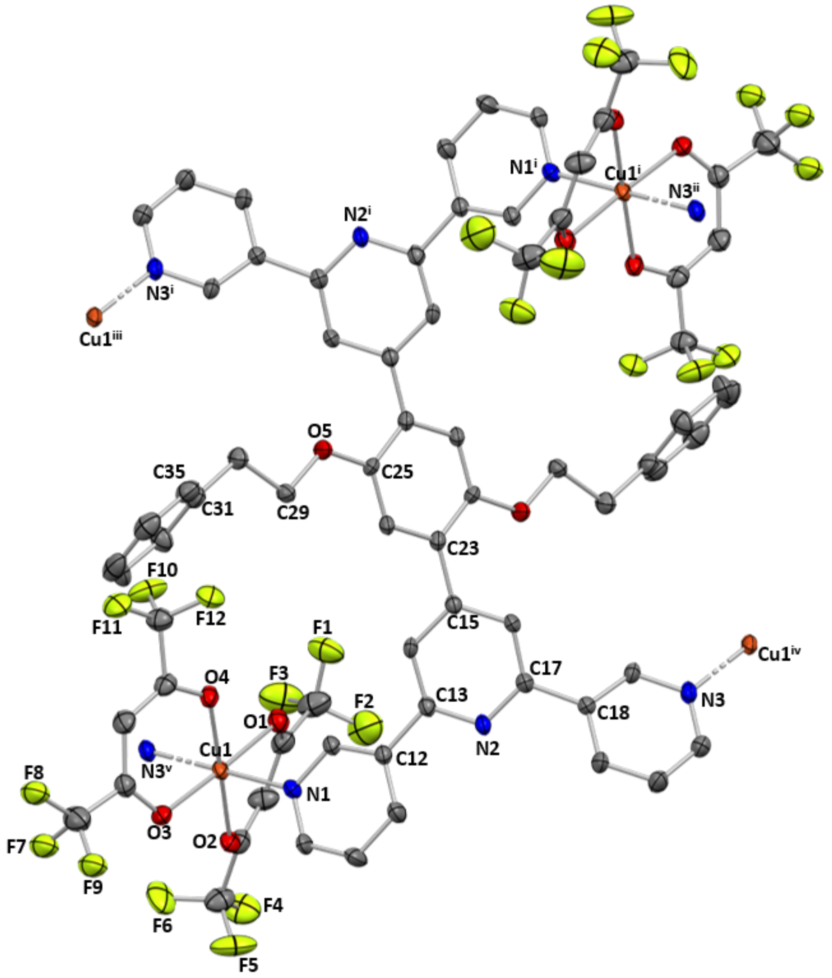

Figure S25. The structure of the asymmetric unit in $[Cu_2(hfacac)_4(\mathbf{1})]_n\cdot 3.6n(1,2\text{-}Cl_2C_6H_4)\cdot 2nCHCl_3$ with symmetry generated atoms. H atoms and solvent molecules are omitted, and ellipsoids are plotted at 40% probability level. Symmetry codes: i = 1–*x*, 1–*y*, 1–*z*; ii = $^1/_2$+*x*, 1.5–*y*, –$^1/_2$+*z*; iii= $^1/_2$+*x*, $^1/_2$–*y*, –$^1/_2$+*z*; iv = $^1/_2$–*x*, $^1/_2$+*y*, 1.5–*z*; v = $^1/_2$–*x*, –$^1/_2$+*y*, 1.5–*z*. The CF_3 group with C10 is disordered and only one of the equal occupancy sites is shown.

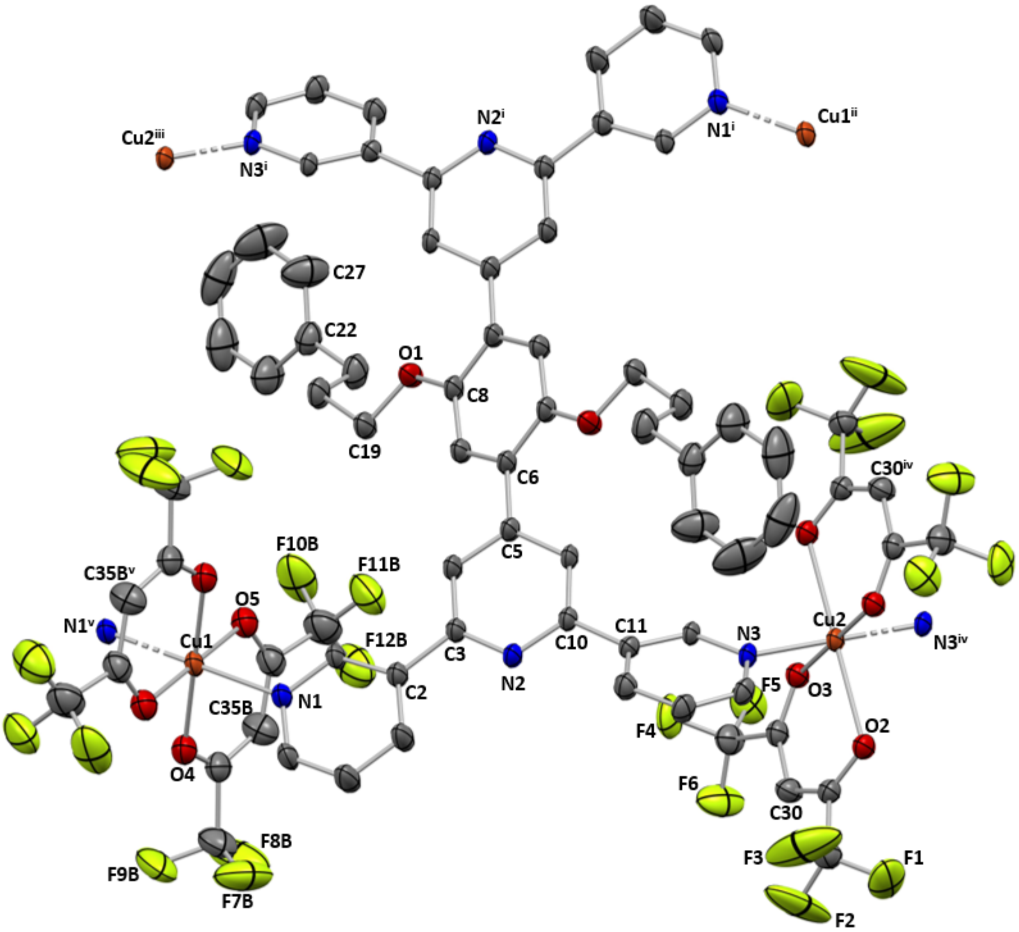

Figure S26. The structure of the asymmetric unit in $[Cu_2(hfacac)_4(\mathbf{2})]_n \cdot 2.8nC_6H_5Cl$ with symmetry generated atoms. H atoms and solvent molecules are omitted, and ellipsoids are plotted at 40% probability level. Symmetry codes: i = 1–*x*, 1–*y*, 2–*z*; ii = *x*, *y*, 1+*z*; iii= 1+*x*, 1+*y*, *z*; iv = –*x*, –*y*, 2–*z*; v = 1–*x*, 1–*y*, 1–*z*. The $[hfacac]^-$ ligand with C35B is disordered and only the major occupancy is shown.

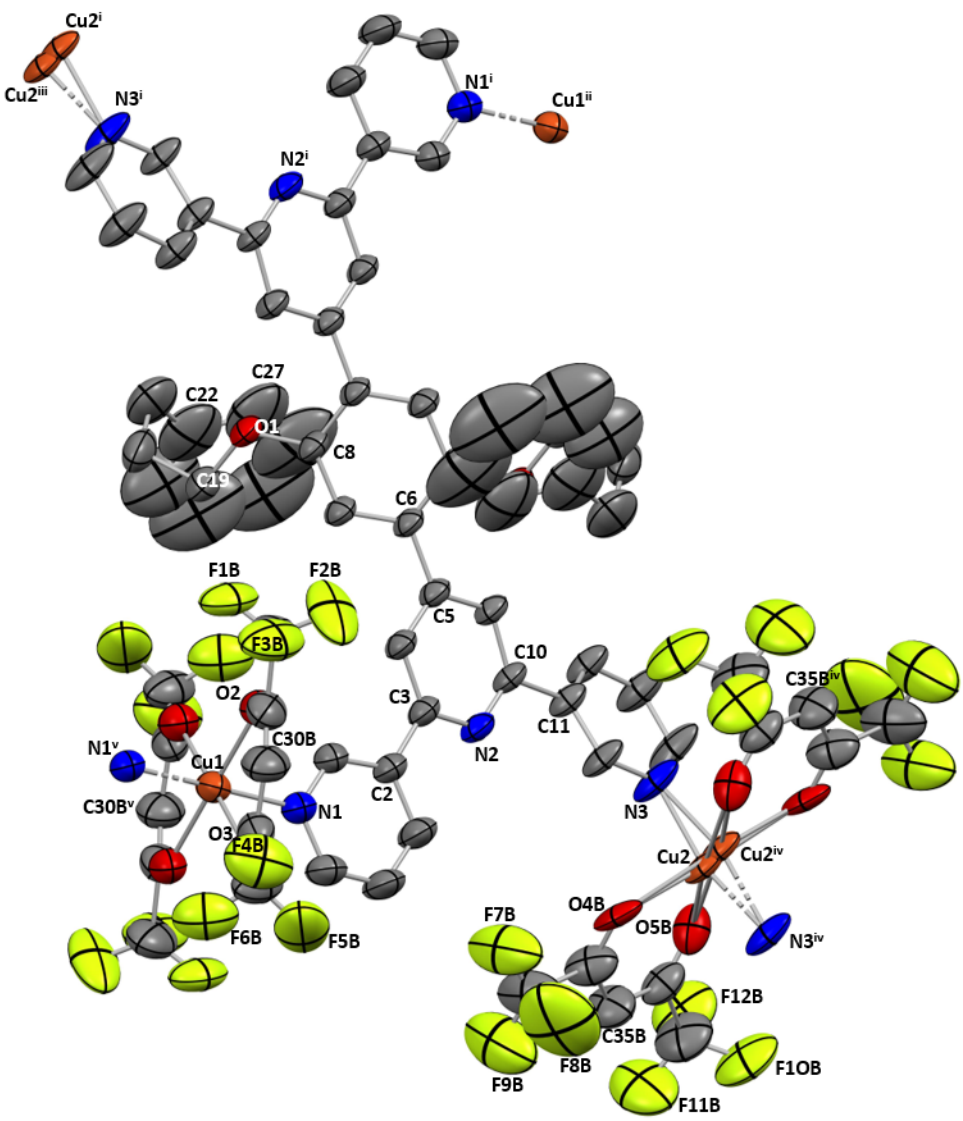

Figure S27. The structure of the asymmetric unit in $[Cu_2(hfacac)_4(\mathbf{2})]_n \cdot 2n(1,2\text{-}Cl_2C_6H_4) \cdot 0.4nCHCl_3 \cdot 0.5nH_2O$ with symmetry generated atoms. H atoms and solvent molecules are omitted, and ellipsoids are plotted at 40% probability level. Symmetry codes: i = –*x*, –*y*, 1–*z*; ii = –1+*x*, –1+*y*, *z*; iii= *x*, –1+*y*, –1+*z*; iv = –*x*, 1–*y*, 2–*z*; v = 1–*x*, 1–*y*, 1–*z*. The $[hfacac]^-$ ligands with C30B and C35B are disordered and only one of the equal occupancy sites is shown.

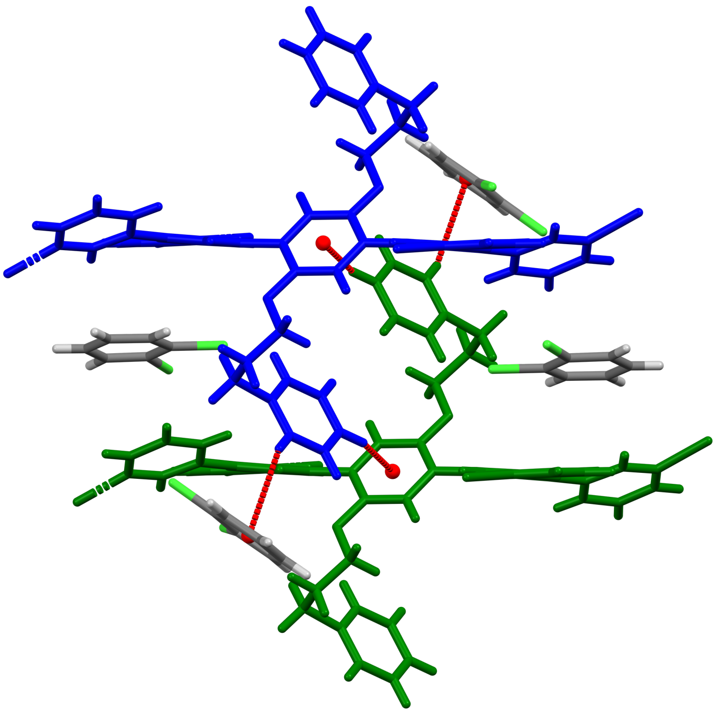

Figure S28. Short C–H...π(arene) interactions are present in $[Cu_2(hfacac)_4(\mathbf{2})]_n \cdot 2n(1,2\text{-}Cl_2C_6H_4) \cdot 0.4nCHCl_3 \cdot 0.5nH_2O$. The contacts arise from the interaction of the peripheral phenyl ring with the 1,2-dichlorobenzene molecule (C–H...centroid separation: 3.16 Å) and the phenylene spacer of the ligand in the adjacent sheet (C–H...centroid separation: 3.00 Å). The two neighbouring 2D-nets are coloured in blue and in green. For clarity, $[hfacac]^-$ ligands are omitted and only one of the equal occupancy disordered sites is shown.

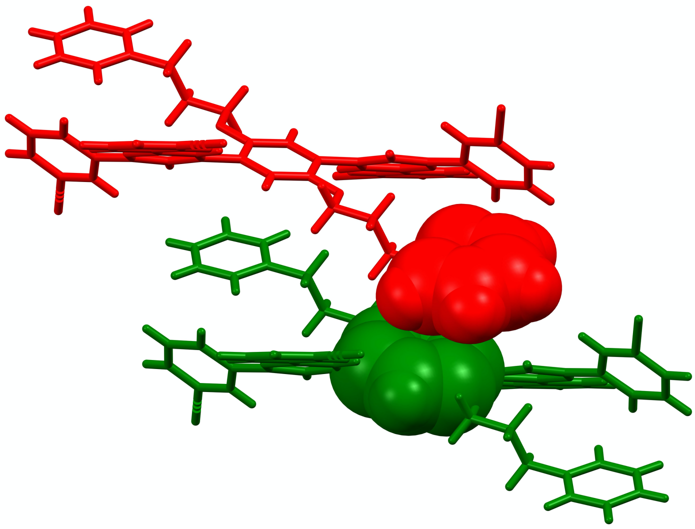

Figure S29. Face-to-face π-stacking interactions are present between the sheets (red and green) in $[Cu_2(hfacac)_4(\mathbf{2})]_n \cdot 2.8nC_6H_5Cl$. The terminal phenyl ring stacks with the central arene spacer of the ligand **2** contained in a neighbouring (4,4)-net. The rings are offset with respect to each other and the centroid...centroid separation is 3.93 Å. For clarity, $[hfacac]^-$ ligands and solvent molecules are omitted, and only the major occupancies of the disordered sites are shown.

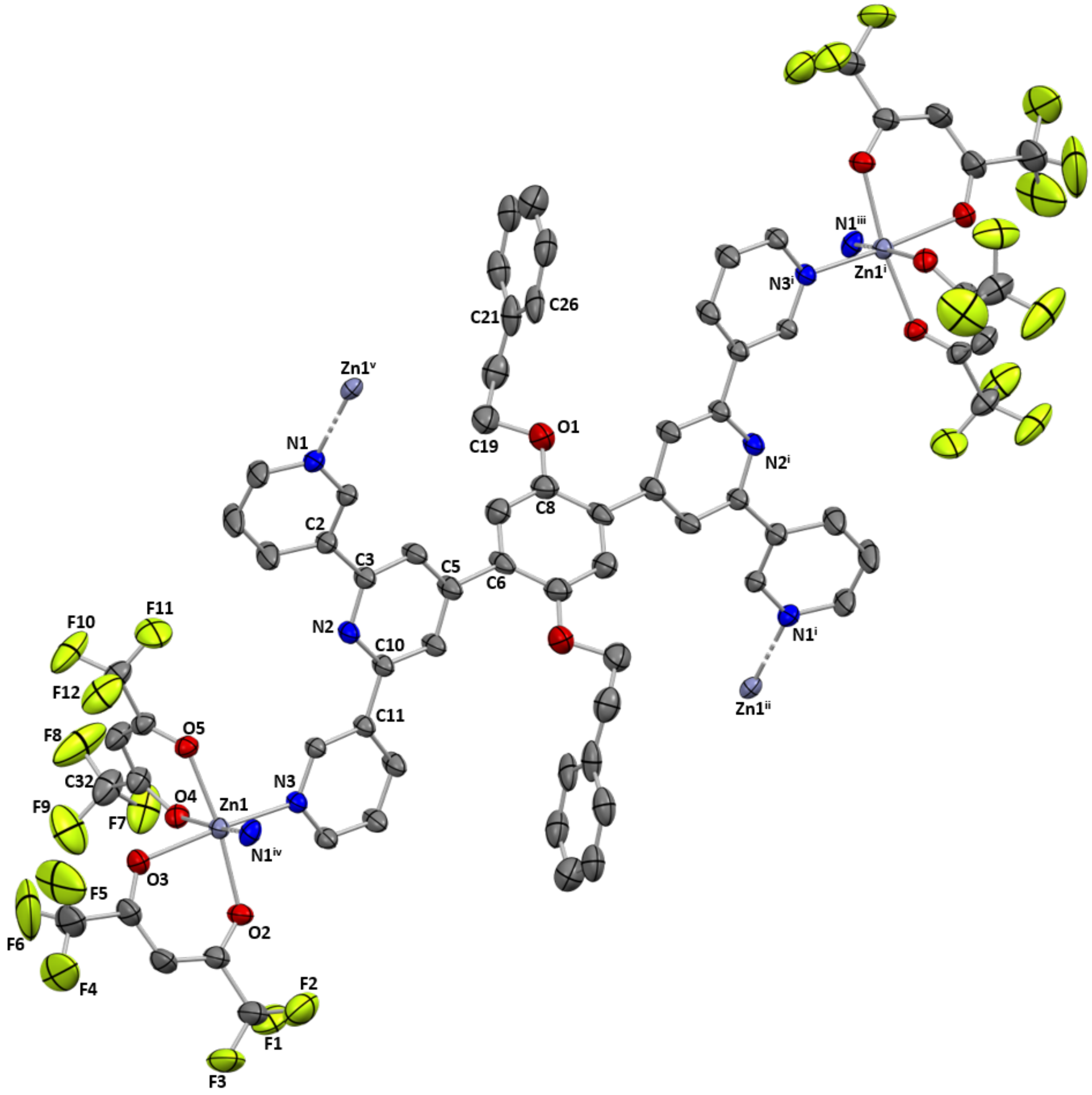

Figure S30. The structure of the asymmetric unit in $[Zn_2(hfacac)_4(\mathbf{1})]_n \cdot n MeC_6H_5 \cdot 1.8 n CHCl_3$ with symmetry generated atoms. H atoms and solvent molecules are omitted, and ellipsoids are plotted at 40% probability level. Symmetry codes: i = 1–*x*, 1–*y*, 1–*z*; ii = $^1/_2$+*x*, 1.5–*y*, $^1/_2$+*z*; iii= $^1/_2$+*x*, $^1/_2$–*y*, $^1/_2$+*z*; iv = $^1/_2$–*x*, $^1/_2$+*y*, $^1/_2$–*z*; v = $^1/_2$–*x*, –$^1/_2$+*y*, $^1/_2$–*z*. The CF_3 group with C32 is disordered and only the major one is shown. The phenylene spacer together with its 3-phenylethoxy substituent is disordered in a 1:1 ratio and only one of the equal occupancy disordered sites is shown.

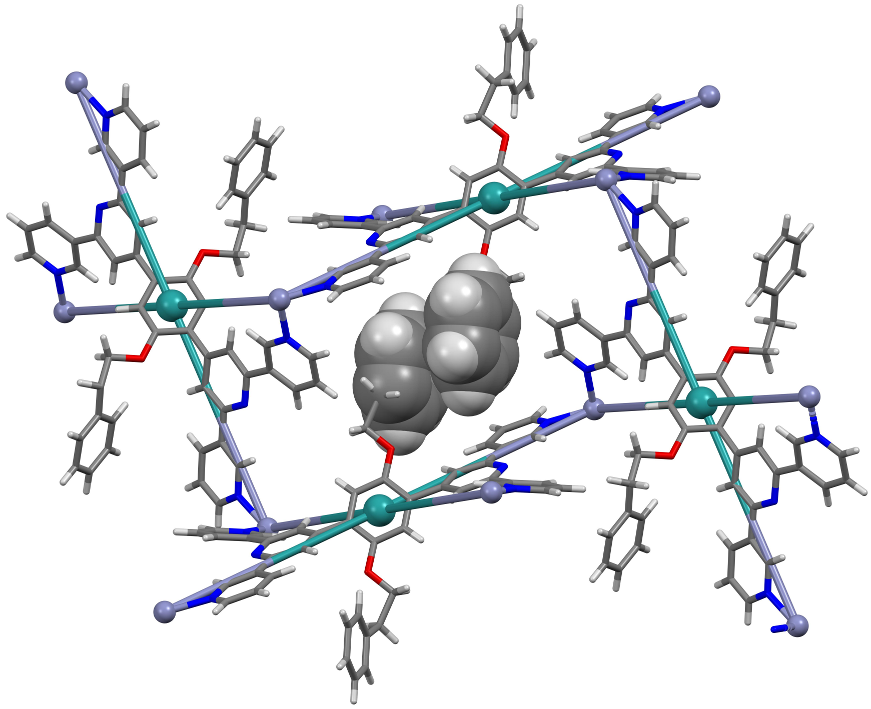

Figure S31. Face-to-face π-stacking interactions are present between pairs of terminal phenyl rings accommodated in the cavities of the (4,4)-net in $[Zn_2(hfacac)_4(\mathbf{1})]_n{\cdot}n MeC_6H_5{\cdot}1.8n CHCl_3$. The rings are offset with respect to each other and the centroid...centroid separation is 4.09 Å. The ligand centroids are coloured in green and define the network. For clarity, $[hfacac]^-$ ligands and solvent molecules are omitted, and only one of the equal occupancy disordered sites is shown.

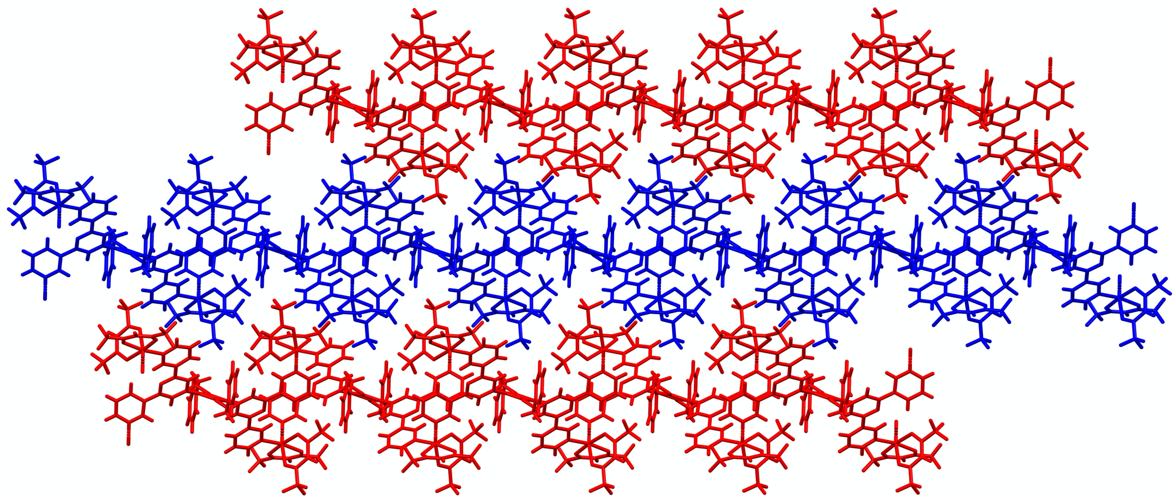

Figure S32. Three layers alternately coloured in red and blue in $[Zn_2(hfacac)_4(\mathbf{1})]_n{\cdot}n MeC_6H_5{\cdot}1.8n CHCl_3$ viewed from the crystallographic *b*-axis. For clarity, solvent molecules are omitted, and only one of the equal occupancy disordered sites is shown.

(a)

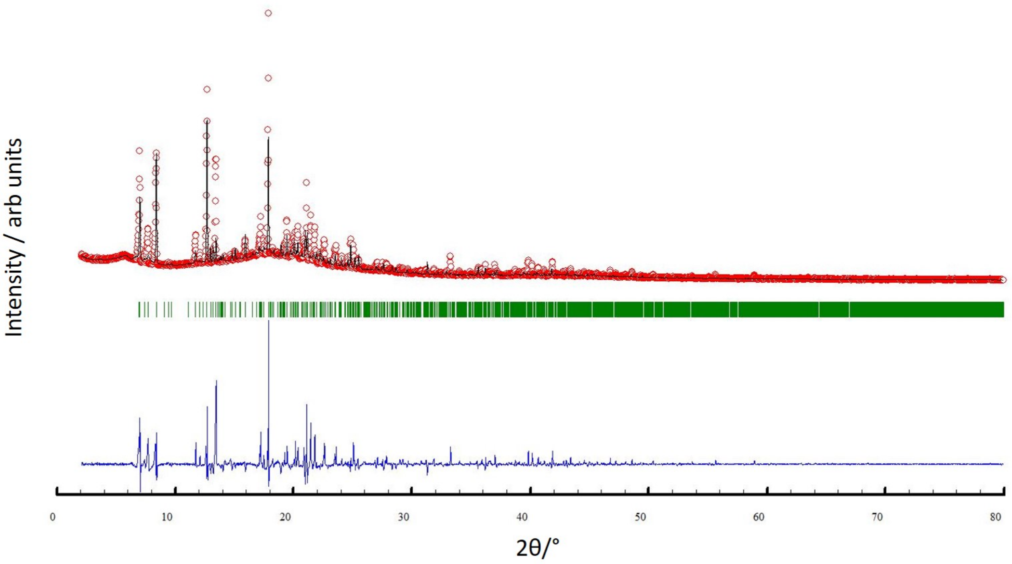

(b)

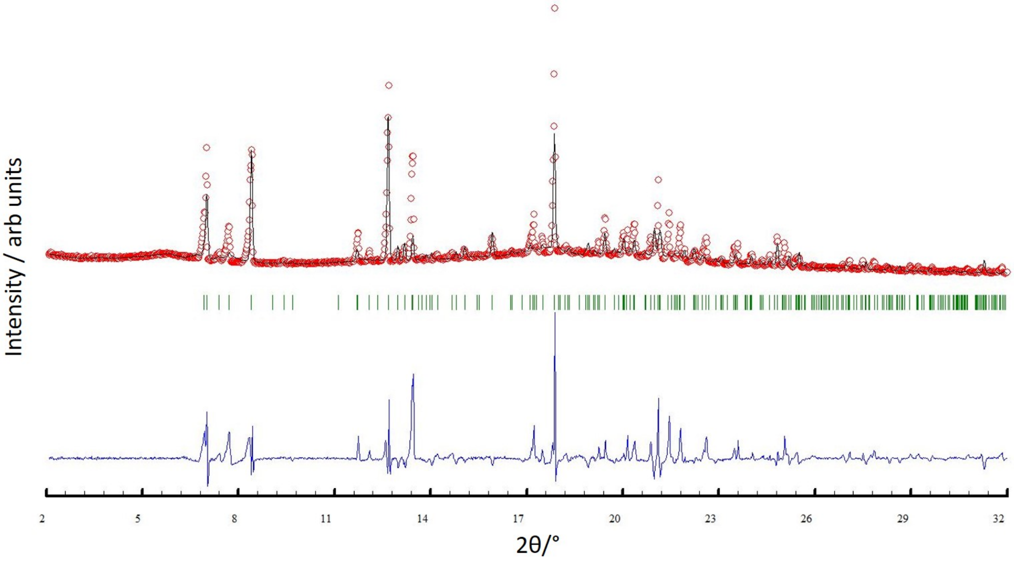

Figure S33. (a) X-ray diffraction (CuKα1 radiation) pattern (red circles) of the bulk crystalline material of $[Zn_2(hfacac)_4(\mathbf{1})]_n{\cdot}n MeC_6H_5{\cdot}1.8nCHCl_3$, fitting the predicted pattern from the single crystal structure. The black lines are the best fit from Rietveld refinements, and green lines display the Bragg peak positions. The blue plot gives the difference between calculated and experimental points. (b) Expansion in the 2-32° range. Preferred orientations of the crystallites explain the differences in the relative intensities of the peaks.

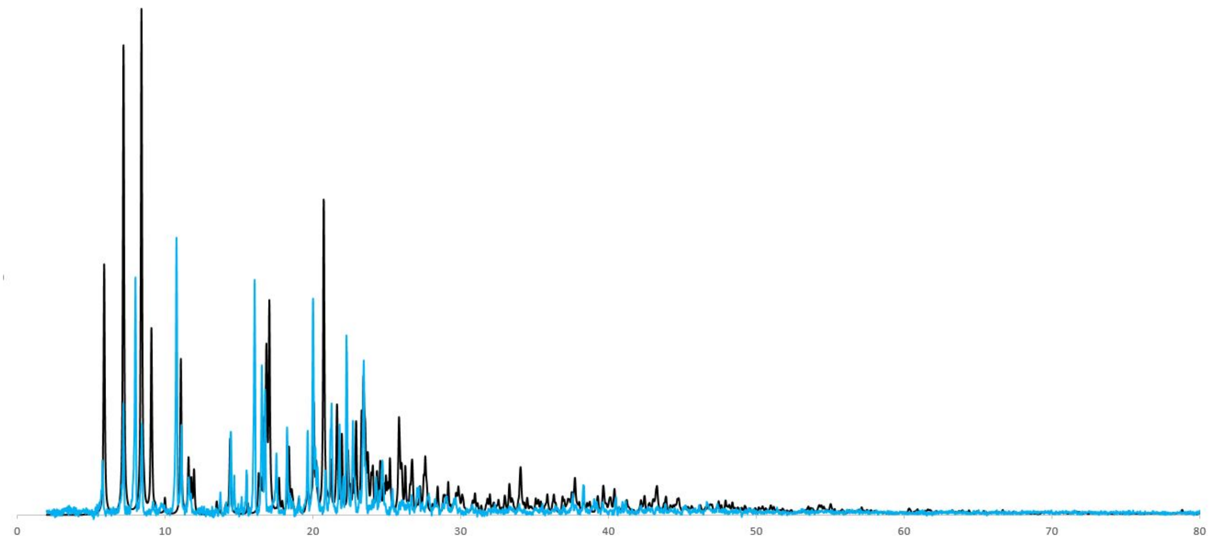

Figure S34. Overlay of the experimental (blue) PXRD (298 K) for the bulk material and that predicted (black) from the single crystal structure (130 K) of $[Cu_2(hfacac)_4(\mathbf{1})]_n \cdot 3.6n(1,2\text{-}Cl_2C_6H_4) \cdot 2nCHCl_3$.

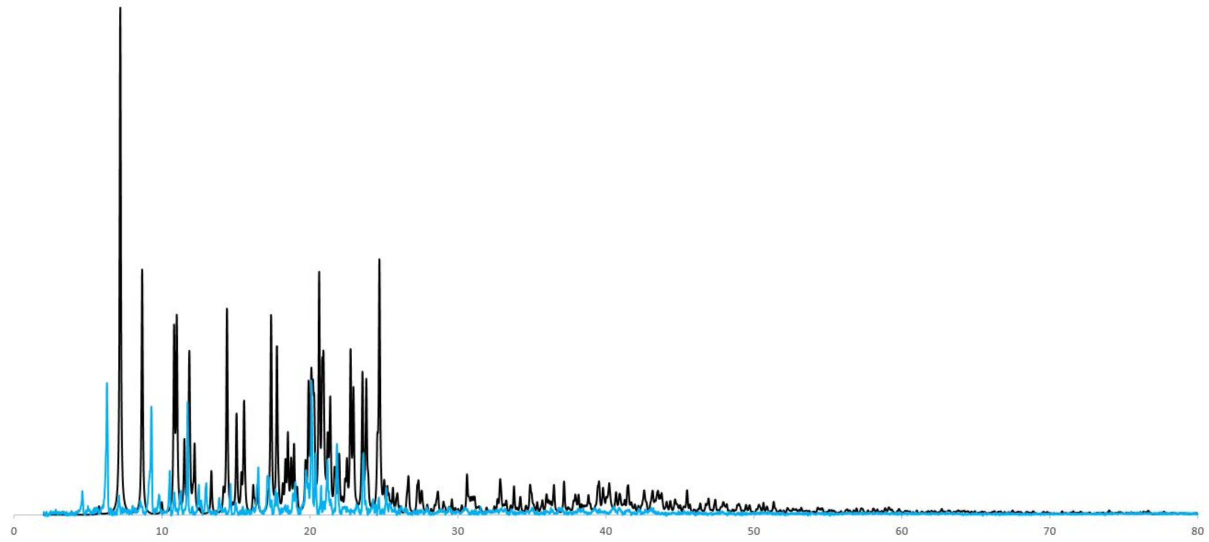

Figure S35. Overlay of the experimental (blue) PXRD (298 K) for the bulk material and that predicted (black) from the single crystal structure (160 K) of in $[Cu_2(hfacac)_4(\mathbf{2})]_n \cdot 2.8nC_6H_5Cl$.

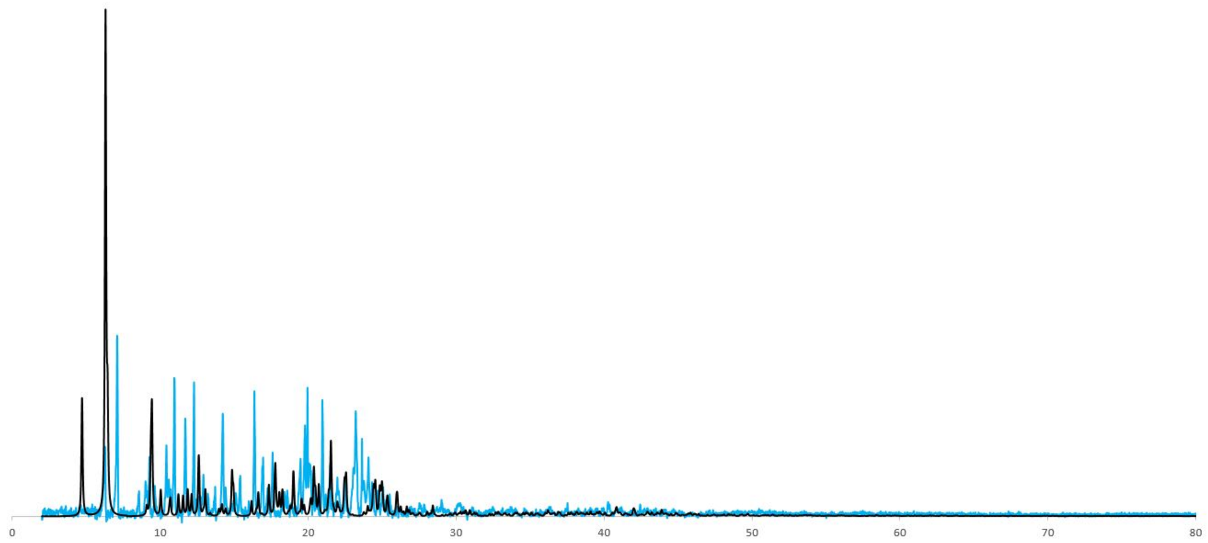

Figure S36. Overlay of the experimental (blue) PXRD (298 K) for the bulk material and that predicted (black) from the single crystal structure (160 K) of in $[Cu_2(hfacac)_4(\mathbf{2})]_n{\cdot}2n(1,2\text{-}Cl_2C_6H_4){\cdot}0.4nCHCl_3{\cdot}0.5nH_2O$.

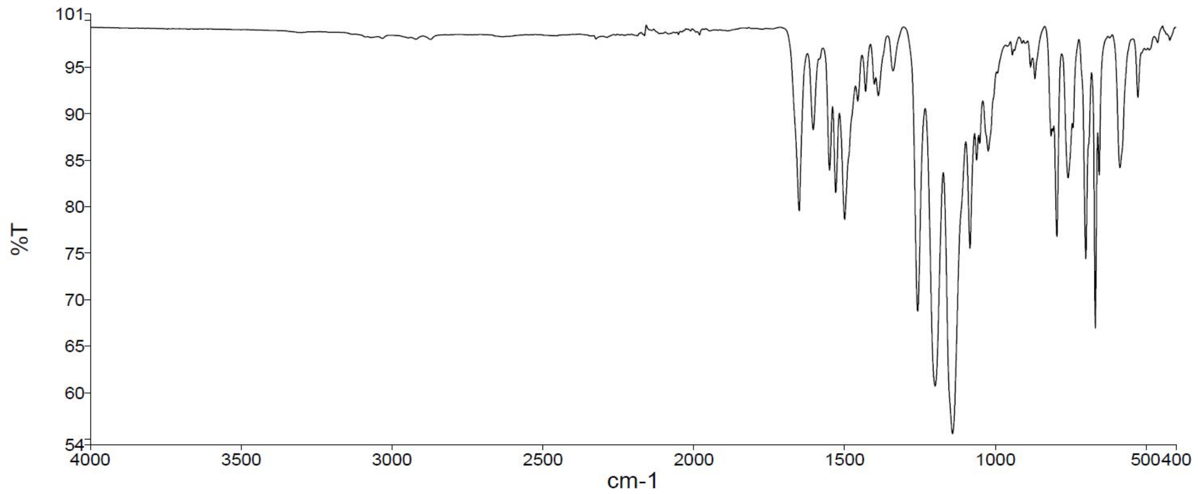

Figure S37. The solid-state FT-IR of $[Cu_2(hfacac)_4(\mathbf{1})]_n{\cdot}3.6n(1,2\text{-}Cl_2C_6H_4){\cdot}2nCHCl_3$.

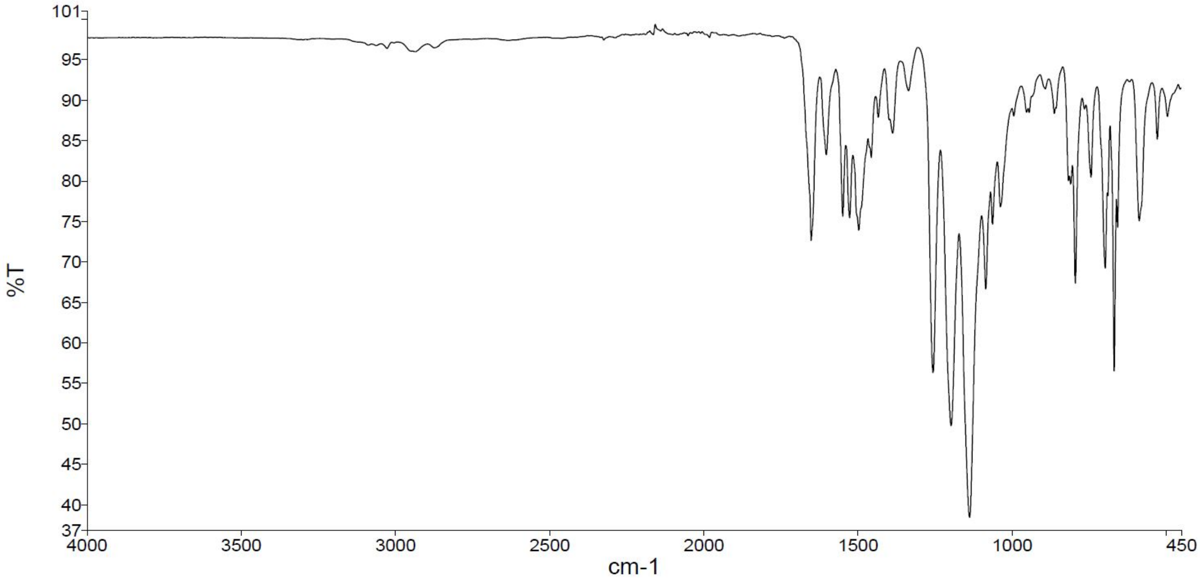

Figure S38. The solid-state FT-IR of $[Cu_2(hfacac)_4(\mathbf{2})]_n{\cdot}nMeC_6H_5{\cdot}2nH_2O$.

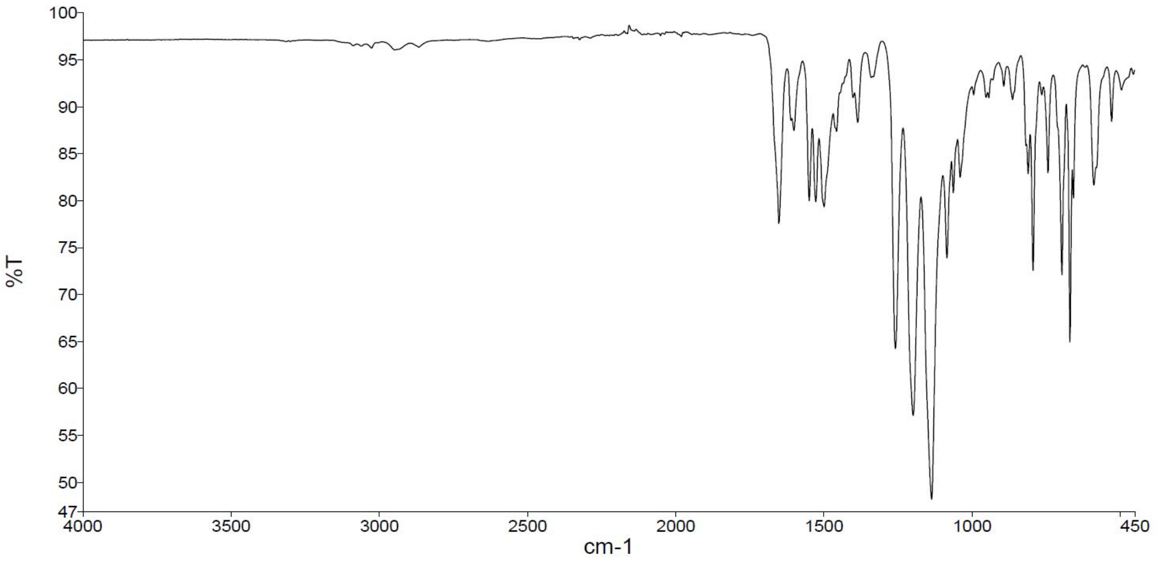

Figure S39. The solid-state FT-IR of [Cu_2(hfacac)$_4$(**2**)]$_n$·2.8$n$$C_6H_5Cl$.

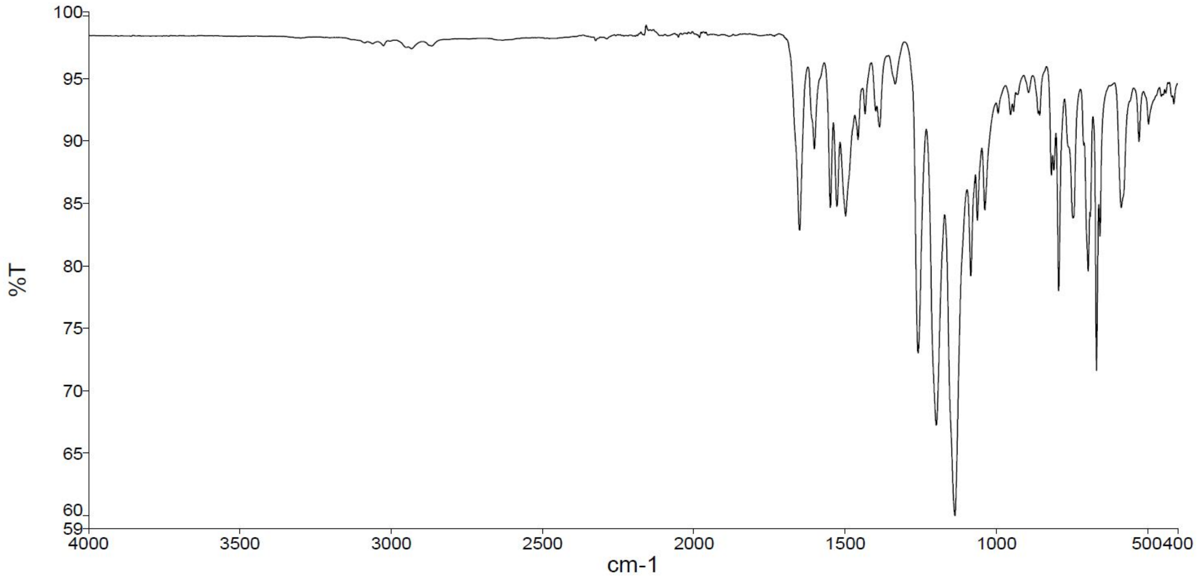

Figure S40. The solid-state FT-IR of [Cu_2(hfacac)$_4$(**2**)]$_n$·2n(1,2-$Cl_2C_6H_4$)·0.4$n$$CHCl_3$·0.5$n$$H_2O$.
